# Network analysis to explore the anti-senescence mechanism of Jinchan Yishen Tongluo Formula (JCYSTLF) in diabetic kidneys

**DOI:** 10.1016/j.heliyon.2024.e29364

**Published:** 2024-04-12

**Authors:** Hongmei Lu, Jing Guo, Yachun Li, Xueqin Zhang, Weijing Liu

**Affiliations:** a Dongzhimen Hospital Affiliated to Beijing University of Chinese Medicine, Beijing University of Chinese Medicine, Beijing, 100700, China; b Key Laboratory of Chinese Internal Medicine of Ministry of Education and Beijing, Beijing, 100700, China; c Zhanjiang Key Laboratory of Prevention and Management of Chronic Kidney Disease, Guangdong Medical University, Zhanjiang, Guangdong, 524001, China; d Hebei University of Chinese Medicine, Shijiazhuang, 050091, China; e Clinical Basic Research Institute of the China Academy of Chinese Medical Sciences, Beijing, 100700, China

**Keywords:** Jinchan yishen tongluo formula, Diabetic nephropathy, Network analysis, HIF-1α, Senescence, Autophagy

## Abstract

**Background:**

The Jinchan Yishen Tongluo Formula (JCYSTLF) has the effect of delaying senescence in diabetic kidneys. However, the mechanism is not clear.

**Purpose:**

Combination methods to investigate the anti-senescence mechanism of JCYSTLF in diabetic kidneys.

**Methods:**

The main compounds of JCYSTLF were characterized by LC-MS/MS, and the anti-senescence targets of JCYSTLF were screened via network analysis. Then, we performed in *vivo* and in *vitro* experiments to validate the results.

**Results:**

The target profiles of compounds were obtained by LC-MS/MS to characterize the primary function of JCYSTLF. Senescence was identified as a key biological functional module of JCYSTLF in the treatment of DN via constructing compounds-target-biological network analysis. Further analysis of senescence-related targets recognized the HIF-1α/autophagy pathway as the core anti-senescence mechanism of JCYSTLF in diabetic kidneys. Animal experiments showed, in comparison with valsartan, JCYSTLF showed an improvement in urinary albumin and renal pathological damage. JCYSTLF enhanced the ability of diabetic kidneys to clear senescence-related proteins via regulating autophagy confirmed by autophagy inhibitor CQ. However, HIF-1α inhibitor 2-ME weakened the role of JCYSLTF in regulating autophagy in diabetic kidneys. Meanwhile, over-expressed HIF-1α in HK-2 cells decreased the levels of SA-β-gal, p21 and p53 induced by AGEs. Upregulated HIF-1α could reverse the blocking of autophagy induced by AGEs in HK-2 cells evaluated by ptfLC3.

**Conclusion:**

We provided in *vitro* and in *vivo* evidence for the anti-senescence role of JCYSTLF in regulating the HIF-1α/autophagy pathway.

## 1 Introduction

Diabetic nephropathy (DN) is the leading course of end-stage renal disease (ESRD) with unsatisfactory treatment and prognosis [1,2]. The main clinical features of DN include persistent proteinuria and progressive structural and functional changes in the kidneys [[3], [4], [5]]. As we all know, both DN and chronic kidney disease (CKD) are senescence-related diseases. Anti-senescence is an important target to delay the progress of DN. However, the anti-senescence strategies are limited, and it is necessary to explore efficient anti-senescence therapies.

Traditional Chinese medicine (TCM) has unique advantages in treating diabetes and related complications due to its characteristics of multiple functions. JCYSTLF is composed of the Cicada flower (Chan Hua), Astragalus (Huang Qi), Scrophulariaceae (Xuan Shen), Curcuma (E Zhu), Silkworm (Jiang Can), Leeches (Shui Zhi), Ghost Arrow Feather (Gui Jianyu), and Achyranthes (Niu Xi), which is widely used in clinical practice for its an effective and safe therapy for DN patients, especially for reducing proteinuria and serum creatinine level. Our previous randomized controlled trial (RCT) research has proved that JCYSTLF has a significant improvement in urinary protein and renal function in DN patients, which provides evidence for JCYSTLF to improve renal injury in diabetes [6]. Moreover, our previous studies have showed that JCYSTLF is related to its anti-aging effect in patients with type 2 diabetes [[7], [8], [9]]. However, little is known about the anti-senescence mechanism of JCYSTLF.

Network pharmacology represents a promising method to discover relevant targets and new drugs for specific diseases [10,11]. Li et al. determined the main active compounds and action mechanisms of ge-gen-qin-lian decoction for the treatment of type 2 diabetes using the network pharmacology method [12]. Guo et al. provided a comprehensive network-based strategy for the systematic discovery of functional synergistic modules that are causal determinants of inflammation-induced tumorigenesis [13]. The core targets predicted by network pharmacology and functional synergistic modules analysis strategy provide significant support for analyzing the anti-senescence mechanism of JCYSTLF for treating DN. To explore the anti-senescence of JCYSTLF in diabetic kidneys, we conducted liquid chromatography tandem-mass spectrometry (LC-MS/MS) of JCYSTLF, network analysis, computational prediction, and experiments to validate the JCYSTLF's anti-senescence effect and molecular mechanism.

## 2 Materials and methods

### 2.1 LC-MS/MS analysis of JCYSTLF

A UHPLC system (Vanquish, Thermo Fisher Scientific) was used to perform LC-MS/MS. The parameters were set, including sample injection volume (5 μL), flow rate (0.5 mL/min), and Waters UPLC BEH (C18 column 1.7 μm 2.1 × 100 mm). The mobile phase contained A and B, where A and B represented 0.1 % formic acid in water and acetonitrile, respectively. The linear elution gradient program was defined (0–11 min, 11–12 min, 12–14 min, 14–14.1 min, 14.1–16 min).

We performed characterization of JCYSTLF via IDA acquisition mode, which was analyzed using Orbitrap Exploris 120 mass spectrometer and Xcalibur software. The range of mass was set from 100 to 1500. The parameters were set, including MS/MS resolution (15000), sheath gas flow rate (35 Arb), ion transfer tube temperature (350 °C), aux gas flow rate (15 Arb), vaporizer temperature (350 °C), spray voltage (4 kV), and full MS resolution (60000).

### 2.2 Target prediction and compounds-target-biological functional module network analysis

Combined with LC-MS/MS, Traditional Chinese Medicine Systems Pharmacology Database (TCMSP), and literature reports identified compounds characterization of JCYSTLF. The OMIN database and TTD database were used to acquire DN-associated target genes, and the YaTCM and ChEMBL databases were used to obtain JCYSTLF-related target genes. To identify the potential targets, a compound-target network was created with Cytoscape 3.7.1 software. The enrichment analysis of Gene Ontology (GO) and Kyoto Encyclopedia of Genes and Genomes (KEGG) was conducted for predicting core targets and signal pathways using the Database for Annotation Visualization and Integrated Discovery and the OmicShare tool. The compounds-target-biological functional module network was built for screening out biological functional modules of JCYSTLF for treating DN via combining the predicted core targets, characterized compounds of JCYSTLF, and enrichment pathway analysis.

### 2.3 Component-target molecular docking

The Standard Delay Format (SDF) structure profile was retrieved from the PubChem database and converted into a Program Data Base (PDB) profile by Open Babel version 2.3.2 software. The receptor protein was then obtained from the Protein Data Bank database. PYMOL version 2.3.4 software and AutoDock Tools version 1.5.6 software were applied for removing water molecules, isolating proteins, and balancing the charges et al. Autodock Vina version 1.1.2 was then used to dock the component-target.

### 2.4 Preparation of JCYSTLF

JCYSTLF is composed of the Cicada flower (Chan Hua), Astragalus (Huang Qi), Scrophulariaceae (Xuan Shen), Curcuma (E Zhu), Ghost Arrow Feather (Gui Jianyu), and Achyranthes (Niu Xi) et al. The Cicada flower was purchased from Zhejiang Fanya Bio-Pharmaceutical Co., Ltd., and the remaining components were obtained from the Chinese Pharmacy of Dongzhimen Hospital. Quality control refers to the established guidelines in the Pharmacopoeia of The People's Republic of China (2010). The traditional Chinese medicine decoction was concentrated to 2 g/mL after being decocted and mixed twice. The dose of the drug in rats was converted according to the body surface area as human clinical dose × equivalent dose coefficient (6.3).

### 2.5 Animals and experimental design

A total of 24 male Sprague-Dawley (SD) rats (7–8 weeks old) weighing 200 ± 20 g were selected. They were bred in an environment with air-conditioning (22–24 °C), humidity (65%–69 %), and a 12-h light/dark cycle. The experimental rats were provided by Beijing Weitong Lihua Laboratory Animal Technology Co., Ltd. and housed in the Animal Room of the Chinese Medicine Pharmacology Laboratory of Dongzhimen Hospital, affiliated to Beijing University of Traditional Chinese Medicine under a specific license [SXXK(Beijing)2020-0013]. This project was approved by the Animal Research Ethics Board of Dongzhimen Hospital affiliated to the Beijing University of Chinese Medicine (Approval No. 20-10), and all operations complied with the regulations of The National Academies Guiding Principles for the Care and Use of Laboratory Animals (8th edition).

Uninephrectomy combined with low-dose streptozotocin (STZ) (50 mg/kg) was used to build the DN model in rats. Blood glucose level aboves 16.7 mmol/L indicated the successful model. We randomly divided the rats into three subgroups: (1) DN group (n = 6): receiving drinking water 2 mL/day by gastric irrigation; (2) ICYSTLF group (n = 6): receiving JCYSTLF 15.1 g/kg/day by intragastric gavage; (3) JCYSTLF + 2-ME group (n = 6): receiving JCYSTLF 15.1 g/kg/day by intragastric gavage and 2-methoxyestradiol (2-ME) 5 mg/kg/day by intraperitoneal injection; (4) JCYSLTF + CQ group (n = 6): receiving JCYSTLF 15.1 g/kg/day by intragastric gavage and chloroquine (CQ) 15 mg/kg/day by intraperitoneal injection; (5) valsartan group: receiving valsartan 8.31 mg/kg/day by intragastric gavage. The intervention cycle was 12 weeks. Twenty-four-hour urine, aortic blood, and kidneys were preserved and used for relevant detection.

### 2.6 Cell culture and experimental groupings

Human renal tubular epithelial cells (HK-2), provided by professor Jochen Reiser, Miami University, USA, were used for in vitro experiments. HK-2 cells were cultured in Dulbecco's modified Eagle medium (DMEM) (Gibco, 11330032). The 8th-10th-generation cells were used for in vitro experiments. In this present study, advanced glycation end products (AGEs) 100 μg/m were used to treat HK-2 cells according to the previous research [14]. HK-2 cells were treated as follows: (1) control group: receiving complete medium with 10 % normal rat serum for 24 h; (2) model group: receiving complete medium with 100 μg/mL AGE-BSA for 24 h; (3) JCYSTLF group: receiving complete medium with 100 μg/mL AGE-BSA and 10 % JCYSTLF serum for 24 h; (4) JCYSTLF + 2-ME group: receiving complete medium with 100 μg/mL AGE-BSA, 10 μmol/L 2-ME, and 10 % JCYSTLF serum for 24 h.

### 2.7 Histology and immunohistochemistry

We used 4 % formaldehyde to fix the kidneys, embedded the specimens in paraffin, and then cut them into 4 mm sections. The sections were stained with periodic acid-Schiff (PAS), hematoxylin and eosin (HE), and Masson trichrome and then cut into 4-μm-thick sections. The levels of protein in the kidneys were detected by immunohistochemistry (IH). Primary antibodies against tumor suppressor gene (p53) (ab131442), cyclin-dependent kinase inhibitor 1A (p21) (ab109520), and Hypoxia-inducible factor-1 alpha (HIF-1α) (NB100-105) were prepared in advance. After treatment with 3 % H_2_O_2_ and goat serum working solution, primary antibody and secondary antibody were added for staining, and then, diaminobenzidine was used to produce a brown color. diaminobenzidine (DBA) working solution (Dako, Denmark) was used for color rendering, and hematoxylin was used for counterstaining. Anymicro DSS™ system was used to collect the images.

### 2.8 Western blot

Western blot (WB) was applied to measure the expression of proteins in tissues and cells. Lysate (protein phosphatase inhibitor cocktail: RIPA = 1:100) was applied to extract proteins, and the BCA protein assay kit (ThermoFisher, TJ272657) was used for protein quantification. Next, 10 % sodium dodecyl sulfate-polyacrylamide gel electrophoresis was applied to separate samples. Gels were transferred to nitrocellulose (NC) membranes (Axygen, Union City) via performing electrophoresis at 4 °C and 90 V for 1 h. The NC membranes were blocked using 5 % nonfat milk and then incubated with primary antibodies, including anti-p53 (Abcam, ab131442), anti-p21 (Abcam, ab109520), anti-sequestosome-1 (p62) (Abcam, ab51243), anti-microtubule-associated protein 1 light chain 3B (LC3B) (Abcam, ab192890), anti-HIF-1α (Novusbio, NB100-105), and anti-β-actin (Abcam, ab3485). TBST was used to wash the NC membranes, and they were incubated with the secondary antibody for 1.5 h. After washing with TBST 3 times, the bands were quantified with ImageJ (National Institutes of Health, Bethesda, MD, USA). β-actin served as a positive control.

### 2.9 Immunofluorescence staining

Immunofluorescence staining (IF) was conducted to detect the expression of proteins in in-vitro experiment sections. After removing the medium, HK-2 cells were washed three times with PBS and then fixed using 4 % fixing solution (1 mL) for 15 min. Next, 0.2 % TritonX-100 solution (1 mL) was added to each well for 10 min. After washing with PBS, 3 % donkey serum (300 μL) was added and incubated for 30 min at room temperature. After discarding the donkey serum, 1.5 % donkey serum (300 μL) was added to dilute the primary antibodies (p62 1:100; LC3 1:1000). Then, the HK-2 cells were placed in a refrigerator at 4 °C overnight. After washing three times with PBS, the secondary antibody was added and incubated for 90 min. After discarding the secondary antibody and washing with PBS, the HK-2 cells were treated with an anti-quenching sealing agent containing DAPI and then placed in a refrigerator at 4 °C overnight. Finally, the average fluorescent strength of each image was calculated with the Image-PRO Plus software (version 6.0) for statistical analysis.

### 2.10 Lentivirus transfection

Lentiviruses carrying the overexpression vector of HIF-1α were purchased from GeneJIKAI (Shanghai, China). The sequences of the HIF-1α overexpression virus are listed in Table S1. A total of 1 × 10^5^ HK-2 cells were transfected with lentiviruses (4.58 × 10^8^ IU/mL, puromy**c**in resistance) for 12 h. After three days, cells were treated with a complete medium that contained 2.0 μg/mL puromycin for 7–10 days to screen stably transfected cell lines.

### 2.11 mRFP-EGFP-LC3B assay

The cells were transfected with mRFP-EGFP-LC3B (5.55 × 10^8^IU/mL, puromycin resistance) (GeneJIKAI, Shanghai, China) for 12h followed by normal media replacement for 48 h. And then, the complete medium with 2.0 μg/mL puromycin was used to screen stably transfected cell lines. Cells with GFP-LC3B positive particles (green), mRFP-LC3B positive particles (red), or GFPC mRFP positive puncta (yellow) were investigated and at least 50 cells/sample were counted three times.

### 2.12 Statistical analysis

IBM SPSS Statistics 22.0 software was used to carry out statistical analysis. The data were expressed as mean ± standard deviation. The Kolmogorov-Smirnov test was applied to examine the normality of data, and the Levene test was applied to test for the homogeneity of variance. If the distribution conformed to the normality and the variance was homogeneous, one-way analysis of variance (one-way ANOVA) was used to compare groups, and the LSD test was applied for pairwise comparisons; if the data did not conform to the normal distribution or the variance was unequal, we chose the Kruskal-Wallis rank-sum test. *P* < 0.05 was considered statistically significant.

## 3 Results

### 3.1 Characterization of JCYSTLF compounds

**Fig. 1:**
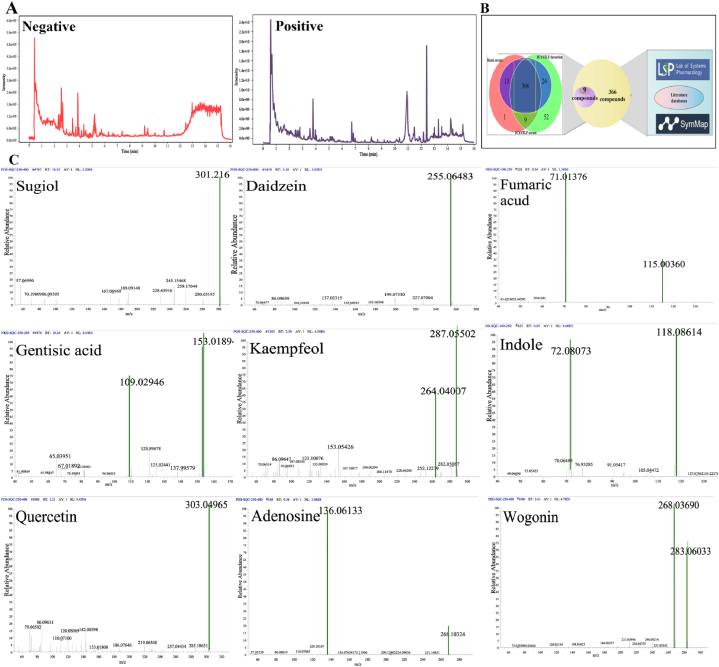
9 compounds of JCYSTLF were identified, including gentisic acid, indole, daidzein, sugiol, quercetin, adenosine, fumaric acid, wogonin, and kaempferol. **(A)** LC-MS/MS analysis result of JCYSTLF. **(B)** Venn diagram of JCYSTLF pharmacochemical analysis based on JCYSTLF decoction, JCYSTLF serum, and blank serum. (**C)** Secondary spectra of 9 compounds.

In this present study, LC-MS/MS was used to characterize the compound information of JCYSTLF. The quality control (QC) of LC-MS/MS is shown in Fig. S1. As shown in Table S1, the quality deviation value (ppm) of compounds identified by LC-MS/MS is less than or equal to 10, indicating a strong reliability of the results. We identified 9 compounds of JCYSTLF by LC-MS/MS, TCMSP database, and literature reports, including gentisic acid, indole, daidzein, sugiol, quercetin, adenosine, fumaric acid, wogonin, and kaempferol (Fig. 1A, B and C). The ppm of 9 compounds is 1.58, 0.829, 4.33, 2.9, 7.09, 4.91, 5.95, 1.24, 4.21, respectively, and the detailed information of these compounds is presented in Table 1.

**Table 1:** Characterization of compounds of JCYSTLF by LC-MS/MS.

No.	Compounds	Pubchem Cid	Class	Score	Formula	mzmed	rtmed	ppm	Adduct
**1**	Daidzein	5281708	flavonoids	0.997557	C15H10O4	255.0648953	186.5845	4.33	[M+H]+
**2**	Gentisic acid	3469	Xanthones	1	C7H6O4	153.019242	619.526	1.58	[M − H]
**3**	Wogonin	5281703	flavonoids	1	C16H12O5	283.0603512	145.459	1.24	[M − H]-
**4**	Indole	798	alkaloid	0.822712154	C8H7N	118.0649025	41.66305	0.829	[M+H]+
**5**	Quercetin	5280343	flavonoids	0.981139385	C15H10O7	301.0371343	208.6095	7.09	[M − H]
**6**	Sugiol	N/A	terpenoids	0.790590615	C20H28O2	301.2151265	623.028	2.9	[M+H]+
**7**	Adenosine	60961	alkaloid	0.838733692	C10H13N5O4	268.1036825	34.85215	4.91	[M+H]+
**8**	Fumaric acid	21883788	Organic acids; derivatives	0.949707846	C4H4O4	115.0036844	33.80325	5.95	[M − H]
**9**	Kaempferol	5280863	flavonoids	1	C15H10O6	285.0399621	252.952	4.21	[M − H]-

### 3.2 Target prediction and compound-target-biological functional module network analysis

**Fig. 2:**
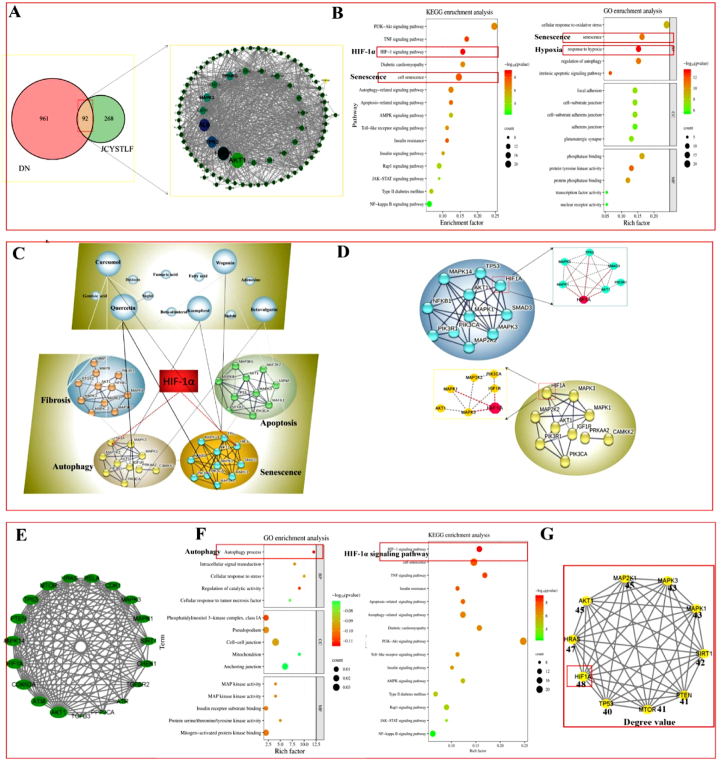
Network pharmacology analysis of the anti-senescence of JCYSTLF in diabetic kidneys. **(A)** A total of 92 targets were identified from DN-related 1114 targets and 440 JCYSTLF-related targets, which were used to construct the PPI network to screen out the core targets of JCYSTLF for treating DN. **(B)** GO signaling pathways were mainly enriched in the biological process of response to hypoxia and regulation of autophagy, and senescence. KEGG signaling pathways were enriched in the HIF-1α signaling pathway, PI3k–AKT signaling pathway, TNF signaling pathway, and autophagy-related and cell senescence-related signaling pathway. **(C)** Network analysis of herb-compound-biological function module. Four key biological functional modules were confirmed, namely, autophagy, cell senescence, fibrosis, and cell apoptosis. **(D)** HIF-1α mainly involved in the biological process of senescence and autophagy. **(E)** The PPI network of senescence-related targets. **(F)** The results of GO enrichment and KEGG enrichment analysis of senescence-related targets. **(G)** The degree value of the anti-senescence targets of JCYSTLF.

As shown in Fig. 2A–a total of 92 targets were selected to construct the protein-protein interaction (PPI) network for screening out core targets. We constructed an essential compound–target–biological function molecule network to uncover the combinatorial rules of JCYSTLF for treating DN from a molecular network perspective based on KEGG and GO enrichment analysis (Fig. 2B). Four key biological functional modules were confirmed, namely, autophagy, cell senescence, fibrosis, and cell apoptosis (Fig. 2C and D), and senescence was predicted as the core mechanism of JCYSTLF in the treatment of DN. We further conducted PPI network of 19 senescence-related targets (Fig. 2E), and identified HIF-1α as the core target of JCYSTLF in anti–diabetic kidneys senescence by constructing KEGG and GO enrichment analysis (Fig. 2F and G). The findings suggests that HIF-1α/autophagy may be the core anti-senescence mechanism of JCYSTLF in diabetic kidneys.

### 3.3 The results of molecular docking

**Fig. 3:**
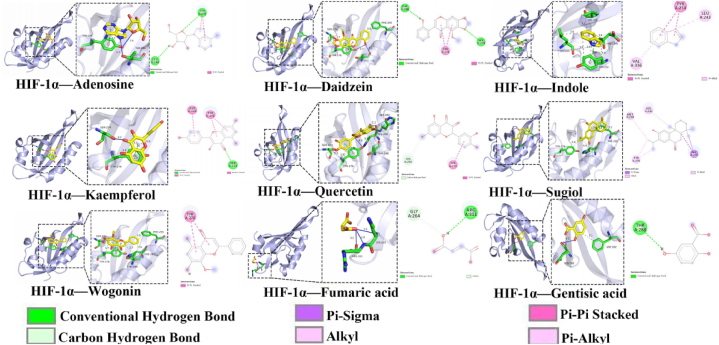
The results of molecular docking.

Molecular docking was used to verify whether the main compounds of JCYSTLF inhibited diabetic kidneys senescence and improved dysfunctional autophagy via targeting HIF-1α to regulate autophagy. As shown in Fig. 3, 9 main compounds of JCYSTLF identified in this present study present a high affinity for HIF-1α and the detailed information is shown in Table 2.

**Table 2:** Representative molecular docking data of the complex of key targets and compounds.

**Target (Uniprot ID)**	**Compounds (Pubchem Cid)**	**Affinity (kcal/mol)**	**Hydrogen Bond**	**Pi-Alkyl**	**Pi -Pi Stacked**	**Pi-Sigma**
HIF-1α (Q16665)	Daidzein (5281708)	−7.0	THR A:288, SER A:274	–	TYR A:276	–
	Gentisic acid (3469)	−4.3	THR A: 288	–	–	–
	Wogonin (5281703)	−6.3	–	–	TYR A:276	–
	Indole (798)	−4.1	–	LEU A: 243, VAL: 336	TYR A:254	–
	Quercetin (5280343)	−7.1	HIS A: 292	–	–	–
	Sugiol (N/A)	−7.3	–	MET A:250	–	PHE A:295
	Adenosine (60961)	−5.2	LEU A: 248, TYR A: 276	–	TYR A: 276	–
	Fumaric acid (21883788)	−4.0	ARG A: 311, GLY A: 264	–	–	–
	Kaempferol (5280863)	−5.9	SER A:276	–	ASP A:249, TYR A: 276	TYR A: 276

### 3.4 JCYSTLF improves proteinuria, renal function, and renal injury in DN rats

**Fig. 4:**
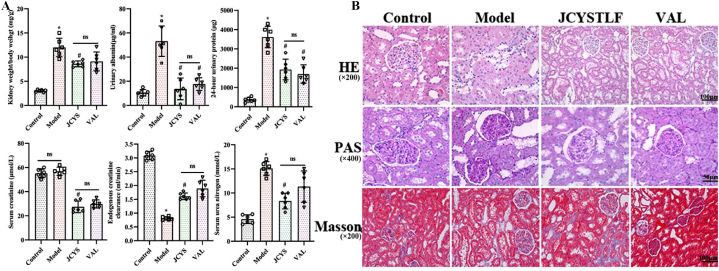
JCYSTLF improves proteinuria, renal function, and renal injury in DN Rats. **(A)** Effect of JCYSTLF on kidney index, urinary protein, and renal function in diabetic rats**. (B)** Effect of JCYSTLF on kidney pathology in diabetic kidneys. Compared with the control group, *P < 0.05, **P < 0.01, and ***P < 0.001; compared with the model group, ^#^*P* < 0.05, ^##^*P* < 0.01, and ^###^*P* < 0.001; renal index: kidney weight/body weight (mg/g).

As shown in Fig. 4A, the kidney index (body weight/kidney weight) was ameliorated after 12 weeks of JCYSTLF treatment (both *P* < 0.05). Compared with the rats in the control group, the rats in the model group developed significant urinary albumin and 24-h urinary albumin, which were significantly reduced after treatment with valsartan or JCYSTLF. Compared with the positive control drug valsartan, JCYSTLF showed an obvious effect on improving urinary albumin in DN rats. There was no significant difference in serum creatinine between the control group and the model group, which may result from the fact that the weight in the model group was lower than that in the control group.

We further analyzed pathological changes in the kidneys. There were significant pathological changes in the model rats, including disruption of the integrity of the tubular structure, thickening of the glomerular basement membrane, extracellular matrix deposition, and renal fibrosis, as shown in Fig. 4B. As expected, there was amelioration after treatment with JCYSTLF or valsartan for 12 weeks. The findings indicates effective renoprotection of JCYSTLF in DN rats.

### 3.5 JCYSTLF clears senescence-related proteins in diabetic kidneys by regulating autophagy

**Fig. 5:**
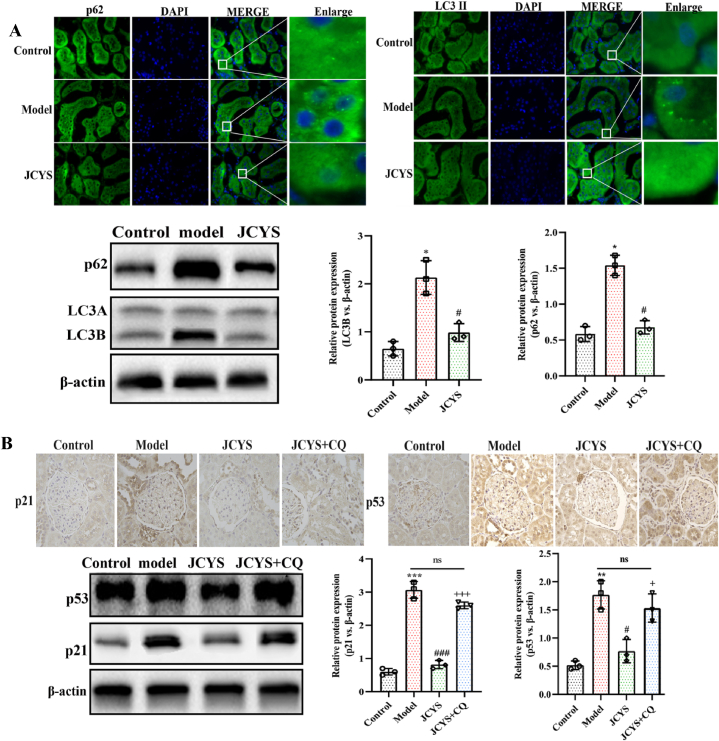
JCYSTLF contributes to clearing senescence-related proteins p53 and p21 via regulating autophagy in diabetic kidneys. **(A)** WB and IF staining for p62 and LCB (Fig.S2–S4). **(B)** The detection of senescence-related protein p21 and p53 via WB and IHC staining (Fig.S5–S7). **P < 0.01 and ***P < 0.001; compared with the model group, ^##^*P* < 0.01 and ^###^*P* < 0.001; compared with the JCYSTLF group, ^++^*P* < 0.01 and ^+++^*P* < 0.001; “ns” represents no statistically significant difference, “JCYS” represents JCYSTLF, and “CQ” represents chloroquine.

Autophagy plays an influential role in maintaining cellular homeostasis. Dysfunctional autophagy is considered the core mechanism of senescence [15,16], which promotes the progression of DN [17,18]. In this study, autophagy was identified as an anti-senescence core mechanism of JCYSTLF in diabetic kidneys. Consistent with previous research findings, JCYSTLF reduced the levels of p53 and p21 in diabetic kidneys (Fig. 5B). Meanwhile, the levels of autophagy-related proteins p62 and LC3B were upregulated in the model group indicating dysfunctional autophagy in diabetic kidneys (Fig. 5A). After treatment with JCYSTLF for 12 weeks, the levels of p62 and LC3B experienced an evident decrease (Fig. 5A). As predicted, the anti-senescence role of JCYSTLF in diabetic kidneys was partially weakened by autophagy inhibitor chloroquine (CQ) (Fig. 5B). The current results shows that JCYSTLF improved senescence in diabetic kidneys probably via regulating autophagy.

### 3.6 JCYSTLF improves dysfunctional autophagy in diabetic kidneys via increasing HIF-1α levels

**Fig. 6:**
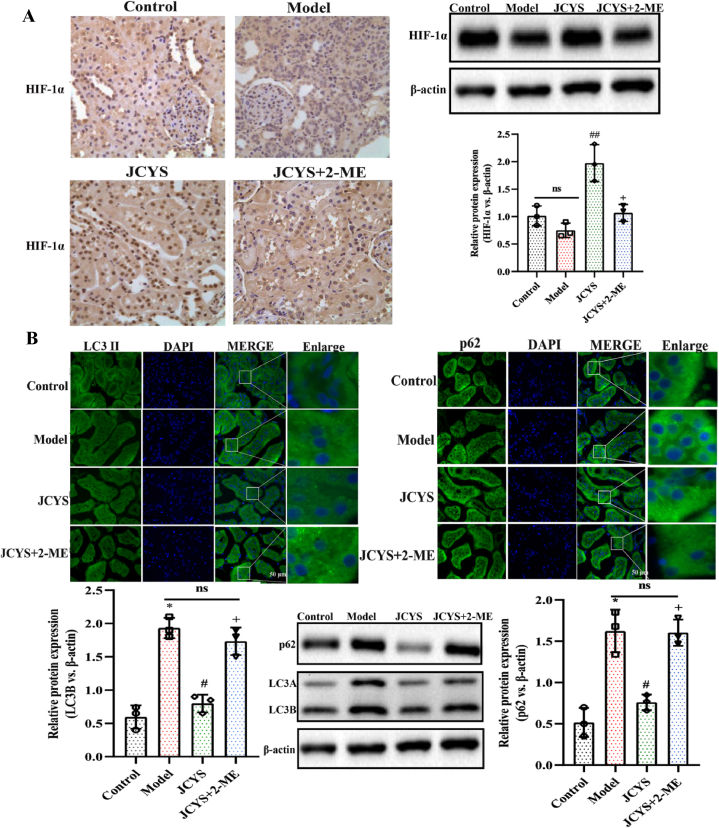
JCYSTLF decreased the levels of p62 and LC3B via increasing the expression of HIF-1α in diabetic kidneys. **(A)** WB and IHC staining for HIF-1α (Fig. S8). **(B)** The evaluation of p62 and LC3B via WB and IF staining (Fig.S9–S11). Compared with the control group, **P < 0.01 and ***P < 0.001; compared with the model group, ^##^*P* < 0.01 and ^###^*P* < 0.001; compared with the JCYSTLF group, ^++^*P* < 0.01 and ^+++^*P* < 0.001; “ns” represents no statistically significant difference, “JCYS” represents JCYSTLF, and “2-ME” represents 2-methoxy estradiol.

HIF-1α is an oxygen-sensitive transcriptional activator in response to hypoxia [19,20], which is widely expressed in kidney cells [21,22]. Recently, HIF-1α has attracted much attention from researchers due to its regulatory effect on autophagy. Based on preliminary confirmation that JCYSTLF improved senescence by regulating autophagy, we introduced HIF-1α inhibitors to further explore whether JCYSTLF regulated autophagy by targeting HIF-1α. As shown in Fig. 6A, we detected the levels of HIF-1α in diabetic kidneys, and the results showed that although there was no obvious difference in HIF-1α levels between the control group and the model group, the levels of HIF-1α in diabetic kidneys increased after treating with JCYSTLF. The relatively low levels of HIF-1α is closely related to DN [23,24], resulting in accelerated kidney senescence [25,26]. Increasing the HIF-1α level enhances the ability of the kidneys to respond to hypoxia, which contributes to improving dysfunctional autophagy [27,28]. Meanwhile, the levels of autophagy-related proteins p62 and LC3B were significantly upregulated in the model group (Fig. 6B). After treatment with JCYSTLF for 12 weeks, the levels of p62 and LC3B experienced a decrease. However, the role of JCYSTLF in improving dysfunctional autophagy was weakened after treatment with 2-ME, indicating that JCYSTLF improved dysfunctional autophagy probably via increasing the levels of HIF-1α.

### 3.7 JCYSTLF improves senescence and autophagy in HK-2 cells induced by AGEs via increasing HIF-1α expression

**Fig. 7:**
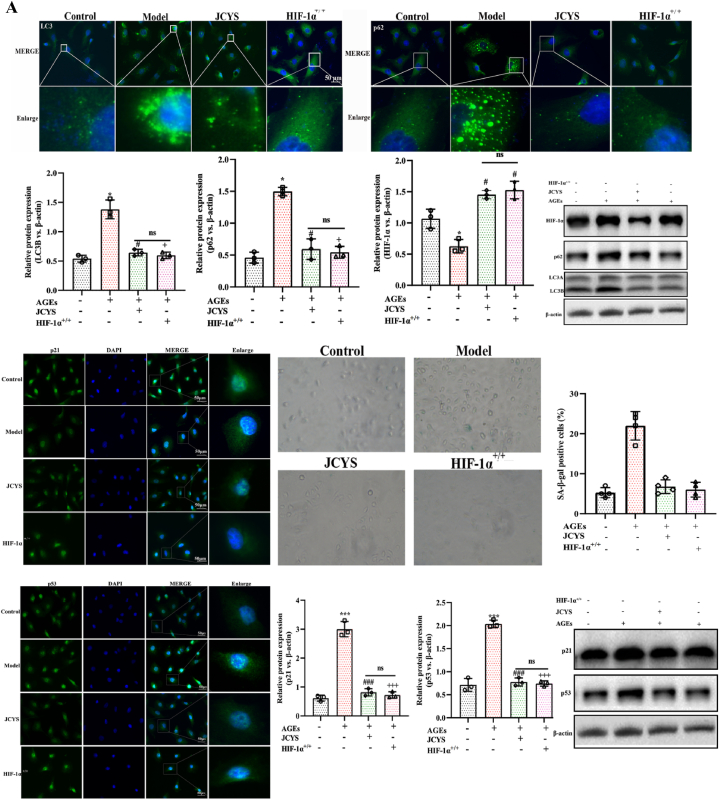

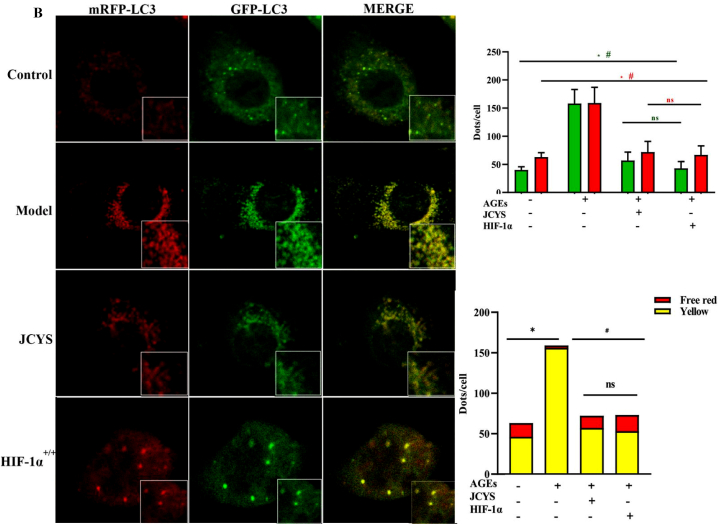
JCYSTLF improved senescence and unblocked autophagy in AGEs-treated HK-2 cells. **(A)** WB and IF staining for p62, LC3B, p21 and p53 (Fig. S12–S17). **(B)** Quantitative analysis of GFP dots (green bars) and mRFP (red bars). The mGFP-RFP-LC3 expression in HK-2 cells AGEs. Points with yellow color in enlarged images represented autophagosomes and points with only red but not green color in enlarged images represented autolysosomes. Compared with the control group, **P < 0.01 and ***P < 0.001; compared with the model group, ^##^*P* < 0.01 and ^###^*P* < 0.001; compared with the JCYSTLF group, ^++^*P* < 0.01 and ^+++^*P* < 0.001; “ns” represents no statistically significant difference, “JCYS” represents JCYSTLF.

As shown in Fig. 7A, compared with the control group, AGEs induced the accumulation of SA-β-gal, p53, and p21 in HK-2 cells, indicating accelerated senescence. The ability of HK-2 cells to clear p53 and p21 was enhanced after treatment with JCYSLT. The expression of HIF-1α was reduced in the model group relative to the control group, which experienced an obvious increase after treating with JCYSTLF. Meanwhile, it was observed that compared with the model group, the levels of SA-β-gal, p21, and p53 were decreased in over-expressed HIF-1α HK-2 cell. The findings shows that JCYSTLF improves HK-2 cells senescence induced by AGEs via upregulating HIF-1α.

As shown in Fig. 7B, the extent of autophagic flux in HK-2 cells was evaluated, and the results showed exposure to AGEs induced the accumulation of p62 and LC3B in HK-2 cells, which experienced a downregulated after treatment with JCYSLTF. Quantitative analysis of autophagosome dots by mGFP-RFP-LC3 expression in AGEs-induced HK-2 cells showed that mGFP-RFP-LC3 expression in HK-2 cells was increased, and the yellow blots were increased. The findings showed that AGEs blocked autophagy flux in HK-2 cells, and JCYSTLF improved autophagy. Meanwhile, over-expressed HIF-1α was proven to unblock autophagy in HK-2 cells treated with AGEs. Overall, JCYSTLF improves autophagy via increasing HIF-1α expression.

## 4 Discussion

As we all know, both type 2 diabetes mellitus (T2DM) and chronic kidney disease (CKD) are senescence-related diseases. It has been reported that in the context of coronavirus disease 2019 (COVID-19) and post-acute COVID-19 syndrome, accelerated renal aging has occurred in patients with diabetes and hypertension [29,30]. Therefore, the prevention and treatment of renal injury in patients with diabetes and hypertension are very important for long-term health [31,32]. In our present study, senescence, as a therapeutic target for treating DN, was identified as one of the key mechanisms of JCYSTLF in the treatment of DN. Current research on JCYSTLF mainly focuses on clinical and experimental validation [33,34]. There have been few reports on the mechanism in diabetic kidneys. We integrated network analysis, literature reports, computational prediction, and in *vitro* and in *vivo* experiments to unveil the mechanism of JCYSTLF for treating DN and recognized some vital active ingredients.

Valsartan, the first-in-class angiotensin receptor-neprilysin inhibitor, could significantly reduce the level of urinary albumin, and alleviate renal function damage in patients with DN [35]. Valsartan is strongly recommended to treat diabetes nephropathy clinically [36]. In the present study, valsartan is used as a positive control drug. Compared with the model group, the rats both in the valsartan group and JCYSTLF group showed obvious improvement in proteinuria, renal function, and pathological damage. Meanwhile, there is no significant difference between the valsartan group and the JCYSTLF group in renal protection of diabetes rats. These findings showes that JCYSTLF has a remarkable renoprotective effect on diabetes rats, providing a basis for exploring the mechanism.

The components of JCYSTLF and related targets of JCYSTLF were measured via LC-MS/MS, TCMSP database, and network analysis. Cell senescence as a vital therapeutic target was screened out by constructing the compound-target-biological functional module network. And, further analysis for senescence-related targets identified HIF-1α/autophagy as the key anti-senescence mechanism of JCYSTLF in the treatment of DN via network pharmacology. A variety of representative ingredients of JCYSTLF have been proven to delay kidney injury in diabetes mellitus. For example, the main active components quercetin [[37], [38], [39]], kojic acid [40], and kaempferol [41] were reported to exert a beneficial action on delaying cell senescence. Our findings also suggests that JCYSTLF has an obvious improvement in senescence in diabetic kidneys, which is consistent with the results of network analysis [42].

HIF-1α is an oxygen-sensitive transcriptional activator in response to hypoxia and plays a pivotal role in physiological and pathological, which involved in senescence, autophagy, oxidative stress et al. [21], [43,44]. Continuous hypoxia is a fundamental internal factor that induces senescence in diabetic kidneys [45,46]. The levels of HIF-1α in diabetic kidneys are lower than those in profound hypoxia, and activation of HIF-1α prevents DN [47,48]. Elevated glucose continuously impairs the stability and function of HIF-1α [15,49], and the impaired HIF-1α may be the vital factor that drives diabetic kidneys persistent senescence [50,51]. HIF-1α activation in the diabetic kidneys may be the target to prevent the senescence of diabetic kidneys [52]. In our study, HIF-1α was predicted as the core target of JCYSTLF for anti-diabetic kidneys senescence and identified part of the key active ingredients related to HIF-1α using molecular docking. According to the results, the anti-senescence effect of JCYSTLF in diabetes is closely related to the upregulation of HIF-1α. Previous research have reported that targeting HIF-1α could be an effective method to ameliorate kidney senescence in patients with diabetes [53,54]. JCYSTLF is found to exert an obvious effect on improving senescence and increasing the levels of HIF-1α.

The main active compound quercetin has been proven to induce HIF-1α protein expression, which is consistent with the results of network prediction and molecular docking. It has been reported that quercetin inhibits cellular proliferation to delay cellular senescence via increasing HIF-1α to inhibit p21 levels [55]. Quercetin is also proven to enhance the responsiveness of epithelial cells to hypoxia via targeting HIF-1α. In our study, we provided evidence both in vivo and in vitro that JCYSTLF exerted renoprotection via promoting HIF-1α protein expression to improve cellular senescence. Quercetin regulates HIF-1α expression in a cell type-specific and intervention conditions-dependent manner. Xu et al. reported that quercetin decreases glucose fluctuation-induced HIF-1α levels in glomerular mesangial cells [56]. Therefore, the regulation of HIF by JCYSTLF may also be different in different renal cells and different exposure conditions, which needs further validation.

HIF-1α/autophagy was identified as a key anti-senescence mechanism of JCYSTLF in the treatment of DN. Autophagy is a complex biological process associated with protein degradation, which is related to senescence. It has been proven that autophagy dysfunction accelerates kidney senescence in a hyperglycemia state, while upregulated HIF-1α has been proven to unblock the autophagy-lysosome pathway [53]. Our previous study have showed significant renal accumulation of p62 and LC3B in patients with DN [57], indicating dysfunctional autophagy in DN patients. Autophagy as a novel therapeutic target for DN has garnered the attention of researchers [58]. In our study, JCYSTLF improves dysfunctional autophagy via increasing HIF-1α. The upregulated HIF-1α increases the response of diabetic kidneys to hypoxia, which contributes to combating hypoxia-induced dysfunctional autophagy [59]. The conclusion is consistent with our research. AGEs mediate kidney cell autophagy abnormality, which weakens the ability to resist senescence and leads to disease progression [60]. JCYSTLF increases HIF-1α levels to unblock autophagy, and over-expressed HIF-1α reverses the dysfunctional autophagy in AGEs-induced HK-2 cells. Current findings shows that JCYSTLF improves dysfunctional autophagy to delay senescence via upregulating HIF-1α. There is emerging evidence that the key active ingredient quercetin [55,61], targets the HIF-1α signaling pathway to regulate the process of autophagy. Moreover, autophagy is considered the center of the senescence process, and mediating autophagy protects renal tubular epithelial cells against accelerated senescence in DN [62].

There are some limitations to the present study. First, certain compounds of JCYSTLF that may produce an effect on DN may have remained unrecognized due to limited detection methods and literature screening. Fortunately, we selected the representative compounds, which have been proven to regulate autophagy and senescence in diabetic kidneys. Second, although certain components were screened as the key active ingredients related to HIF-1α, their experimental validation is lacking. Third, there are still some shortcomings in the experiment section due to the limited types of cells and animal models.

## 5 Conclusion

**Fig. 8:**
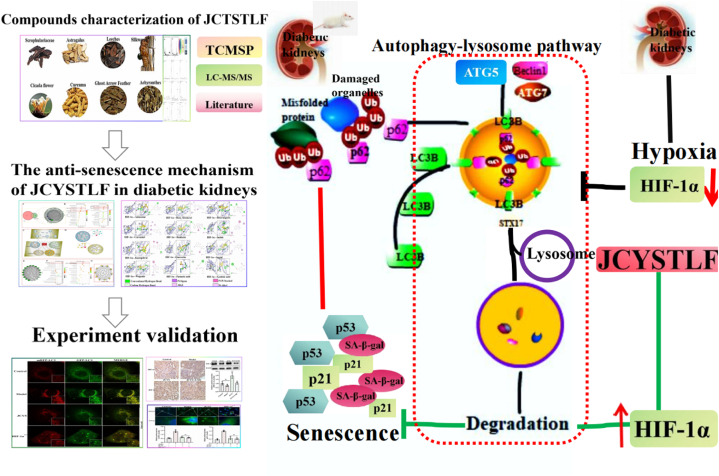
JCYSTLF exerted an anti-senescence effect through regulating the HIF-1α/autophagy pathway in diabetic kidneys. Combined with LC-MS/MS, TCMSP, literature reports, network analysis, and experiment validation identified compounds characterization of JCYSTLF and explored JCYSTLF's anti-senescence mechanism. Misfolded proteins and damaged organelles are degraded mainly by autophagy lysosome pathway. In diabetic kidneys, dysfunctional autophagy induces the accumulation of misfolded proteins and damaged organelles, and accelerates kidney senescence. JCYSTLF increases the levels of HIF-1α to improve dysfunctional autophagy, and delays diabetic kidneys senescence.

In summary, based on network pharmacology, molecular docking, systematic prediction, and experimental validation, JCYSTLF was found to exert an anti-senescence effect through regulating the HIF-1α/autophagy pathway (Fig. 8), which provides evidence for targeting HIF-1α to delay senescence in the diabetic kidneys.

## Ethical approval

This project was approved by the Animal Research Ethics Board of Dongzhimen Hospital affiliated to the Beijing University of Chinese Medicine (Approval No. 20-10).

## Funding

The National Nature Science Foundation of China (No. 82004196, No. 82205035, 82205037, and 82104820) supported this study.

## Data availability statement

The datasets used in this study are available from the corresponding author upon reasonable request.

## CRediT authorship contribution statement

**Hongmei Lu:** Writing – original draft, Validation, Methodology. **Jing Guo:** Visualization, Methodology. **Yachun Li:** Writing – review & editing, Validation. **Xueqin Zhang:** Validation, Funding acquisition, Conceptualization. **Weijing Liu:** Data curation, Conceptualization.

## Declaration of competing interest

The authors declare that they have no known competing financial interests or personal relationships that could have appeared to influence the work reported in this paper.
