# Supplementary material for: Network analysis to explore the anti-senescence mechanism of Jinchan Yishen Tongluo Formula (JCYSTLF) in diabetic kidneys
Source: Heliyon. 2024 Apr 12;10(9):e29364. doi: 10.1016/j.heliyon.2024.e29364 (PMC11076649; doi:10.1016/j.heliyon.2024.e29364)
Supplement: Multimedia component 1 [file mmc1.pdf]

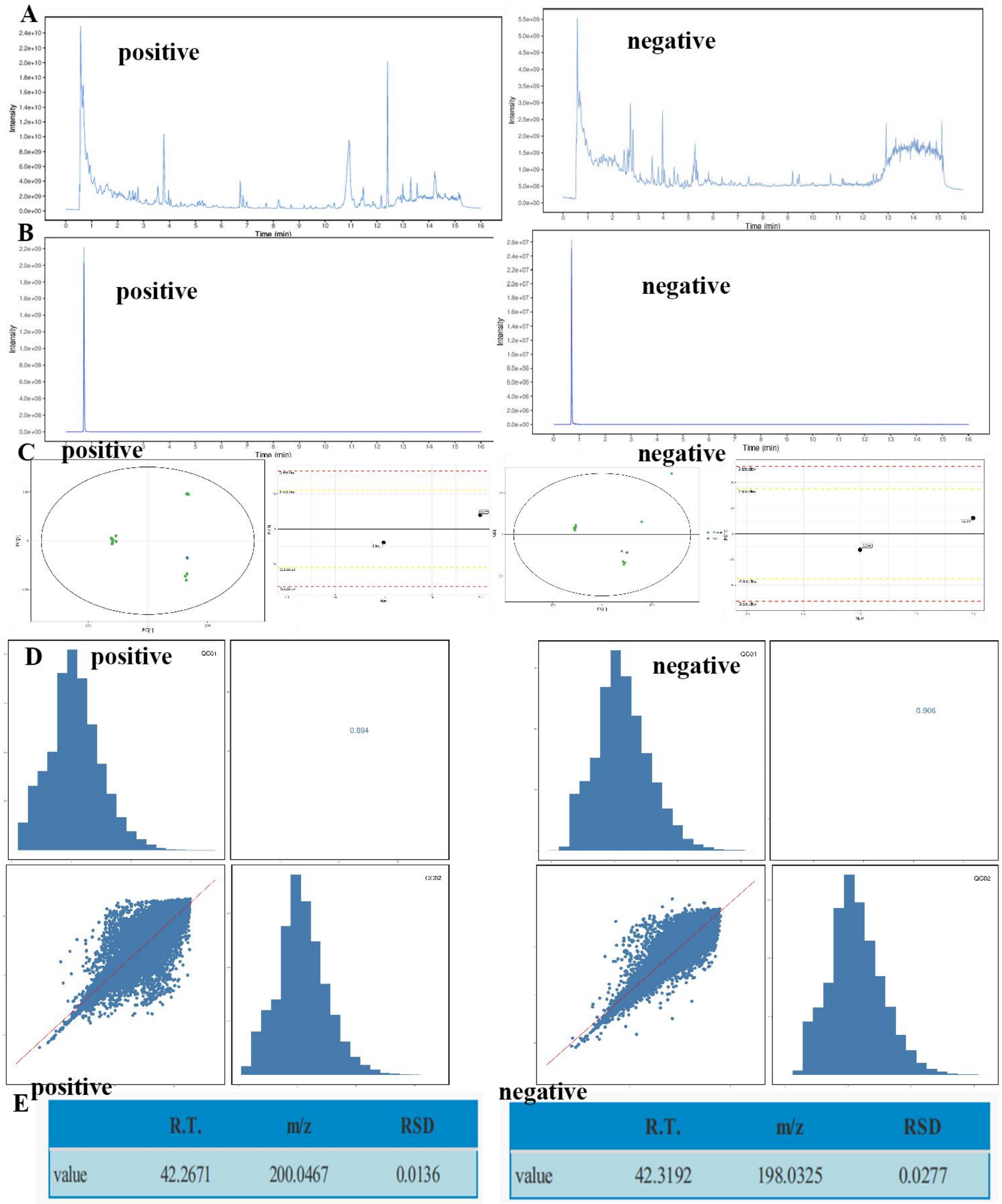

E

positive

	R.T.	m/z	RSD
value	42.2671	200.0467	0.0136

negative

	R.T.	m/z	RSD
value	42.3192	198.0325	0.0277

FigS1: QC of LC-MS/MS. (A) Positive and negative TIC charts of all QC sample. (B) Response of internal standard L-2-chlorophenylalanine of all QC sample. (C) Positive and negative PCA score chart annotation. The blue dots represent QC samples, and the green dots represent formal experimental samples. (D) Positive and negative QC sample correlation. (E) Response stability of QC samples in internal standards.
