# Supplementary material for: Network analysis to explore the anti-senescence mechanism of Jinchan Yishen Tongluo Formula (JCYSTLF) in diabetic kidneys
Source: Heliyon. 2024 Apr 12;10(9):e29364. doi: 10.1016/j.heliyon.2024.e29364 (PMC11076649; doi:10.1016/j.heliyon.2024.e29364)
Supplement: Multimedia component 2 [file mmc2.doc]

| **TableS1:** The ppm value of compounds of JCYSTLF recognized by LC-MS/MS. | | | | | |
| --- | --- | --- | --- | --- | --- |
| **NameEN** | **ppm** | **NameEN** | **ppm** | **NameEN** | **ppm** |
| 2,3-DIHYDROXYBENZOATE | 1.81282461735565 | 9-Hydroxy-10,12,15-octadecatrienoic acid | 2.51814997433237 | (+/-) Salsolinol | 3.19140628675479 |
| 4-[2-(2,6-dimethoxy-4-prop-2-enylphenoxy)-1-hydroxypropyl]-2-methoxyphenol | 0.270395114966694 | 14-hydroxy-14-(hydroxymethyl)-5,9-dimethyltetracyclo[11.2.1.0¹,¹⁰.0⁴,⁹]hexadecane-5-carboxylic acid | 4.02815855921962 | (3beta,5alpha,9alpha,22E,24R)-5,9-Epidioxy-3-hydroxyergosta-7,22-dien-6-one | 2.59299651637938 |
| 5,7-dihydroxy-2-(4-hydroxyphenyl)-8-[3,4,5-trihydroxy-6-(hydroxymethyl)oxan-2-yl]-6-(3,4,5-trihydroxyoxan-2-yl)chromen-4-one | 0.761200097109102 | 3-Hydroxy-5-isopropylidene-3,8-dimethyl-2,3,3a,4,5,8a-hexahydro-6(1H)-azulenone | 3.28314183526636 | 1-Phenanthrenecarboxylic acid, 7-ethenyl-1,2,3,4,4a,4b,5,6,7,9,10,10a-dodecahydro-9-hydroxy-1,4a,7-trimethyl- | 0.560998607573406 |
| 9-hydroxy-10,12-octadecadienoic acid | 1.92015096362551 | FA 18:1+1O | 0.236407324545531 | Aleuritic acid | 0.460794014264263 |
| Coumaric acid | 1.02504257422949 | 3,4,5-trimethoxycinnamic acid | 2.2185855425273 | Suberic acid | 2.24282869138223 |
| Curcumenol | 0.666274576030235 | 7-Hydroxycoumarin | 3.38520024786994 | Kaempferide | 1.30117753625075 |
| 7-methoxy-6-(1,2,3-trihydroxy-3-methylbutyl)chromen-2-one | 10.9854709860152 | 2,8-Dihydroxy-5,5,8-trimethyl-11-oxatetracyclo[7.3.1.0~1,9~.0~3,7~]tridecan-10-one | 0.813700482159915 | 2-[[(2R,3S)-2-(3,4-dihydroxyphenyl)-3,5-dihydroxy-3,4-dihydro-2H-chromen-7-yl]oxy]oxane-3,4,5-triol | 2.50743148752977 |
| Emodin | 2.61547553186583 | Methyl gallate | 5.01536313423611 | 2'',4''-Diacetylafzelin | 7.20882609189924 |
| Gentisic acid | 1.58137273364608 | Glyceric acid | 3.18984426073853 | 3-Hydroxykynurenine | 4.45526514494741 |
| 7-hydroxy-1,4a-dimethyl-9-oxo-7-propan-2-yl-2,3,4,4b,5,6,10,10a-octahydrophenanthrene-1-carboxylic acid | 0.838832781519151 | 5-hydroxy-2,2-dimethyl-10-(2-methylbut-3-en-2-yl)pyrano[3,2-g]chromen-8-one | 3.16450317171657 | 3-(2-methylpropyl)-2,3,6,7,8,8a-hexahydropyrrolo[1,2-a]pyrazine-1,4-dione | 23.2480055921429 |
| Kaempferol | 0.13285149119573 | Flavonol base + 3O, O-Hex-Hex | 1.65596214792767 | 4-Aminobenzoic acid | 1.17191923942204 |
| Liquiritin | 0.906760909498712 | Linoleic acid | 0.92389739588605 | 4-Methoxysalicylic acid | 1.79094796053881 |
| Nicotinic acid | 1.72650792655193 | Pinocembrin | 0.601630359716981 | Isokobusone | 1.15685739811527 |
| p-Hydroxybenzaldehyde | 4.42246441670809 | FA 9:1+1O | 2.69427668255653 | 5,7-dihydroxy-2-(4-hydroxyphenyl)-6,8-bis(3,4,5-trihydroxyoxan-2-yl)chromen-4-one | 2.40670699329836 |
| Quebrachitol | 0.861001233368518 | Atractylenolide III | 1.13161099699702 |  |  |
| STACHYOSE | 5.7865225007117 | Saikosaponin b2 | 6.99073979904662 | 7-Hydroxycoumarin | 0.884105100198509 |
| Trans-Vaccenic acid | 32.0946531172038 | Zizyberanalic acid | 1.63827562968332 | 7-Methoxycoumarin | 2.74936296073407 |
| Wogonin | 1.24086777409457 | Ethylparaben | 2.27486976351027 | 8-Desoxygartanin | 2.2592565424859 |
| Formononetin | 2.41678113973542 | Nifedipine | 1.21735809528861 | Acetic anhydride | 2.40423244202516 |
| Hydroxygenkwanin | 4.27376850321143 | Chaulmoogric Acid | 0.344531371829398 | Adenine | 3.76409230578903 |
| Genkwanin | 0.30694591043165 | Oleanane -2H, +1O, 1COOH, O-HexA-HexA | 1.13743746452896 | Allixin | 2.93819292382193 |
| 4-hydroxybenzoic acid | 2.10394633386033 | SUCROSE | 1.36401788973052 | Apigenin | 1.93544377038759 |
| MANNITOL | 5.15676426863494 | (EZ)-sinapic acid | 1.16775866775014 | Azuleno(5,6-c)furan-1(3H)-one, 4,4a,5,6,7,7a,8,9-octahydro-3,4,8-trihydroxy-6,6,8-trimethyl- | 2.41522990925436 |
| isosakuranetin-7-O-rutinoside | 3.96567654523753 | Glutamylphenylalanine | 0.500026887250752 | Benzoic acid | 0.257039921698469 |
| FA 18:1+3O | 1.07016411807199 | Kainic acid | 3.39968996303395 | Beta-Caryophyllene Alcohol | 1.10846389375861 |
| Abscisic acid | 1.43928319270578 | alpha,beta-Dihydroresveratrol | 0.486426659823086 | Bullatine A | 10.3816557201571 |
| Dihydrokaempferol | 2.28219994725644 | 3-coumaric acid | 0.757146169473189 | Calycosin | 3.0836872057375 |
| Azuleno(5,6-c)furan-1(3H)-one, 4,4a,5,6,7,7a,8,9-octahydro-3,4,8-trihydroxy-6,6,8-trimethyl- | 0.282274691135405 | 2,4,6-trihydroxy-5-[1-(4-hydroxy-1,1,4,7-tetramethyl-1a,2,3,4a,5,6,7a,7b-octahydrocyclopropa[h]azulen-7-yl)-3-methylbutyl]benzene-1,3-dicarbaldehyde | 1.93158721293663 | 3,8a-Dihydroxy-5-isopropylidene-3,8-dimethyl-2,3,3a,4,5,8a-hexahydro-6(1H)-azulenone | 0.752408757423015 |
| Quercetin-3-O-galactoside | 2.23095678602758 | 2-Phenylethyl octanoate | 2.14999981083823 | Choline [M]+ | 2.47146528953735 |
| L-ISOLEUCINE | 2.51772926874939 | trans-Ferulic acid | 1.18977963688557 | Chrysin Dimethyl Ether | 2.93907740658386 |
| 13-HODE | 1.42485827737266 | Ethyl 4-methoxycinnamate | 0.582812123867836 | Cinnamyl alcohol | 2.65880782550277 |
| Soyasapogenol E base + O-HexA-Hex-dHex | 0.949022461878089 | Indole | 0.825645741640229 | Diethyl malonate | 1.50742154318816 |
| Citric acid | 1.73796666824377 | Chrysin | 1.53383659213848 | Diethyltoluamide | 1.33652306039914 |
| 2-AMINOPHENOL | 4.86552961453109 | Isoyatein | 5.40330826056923 | Dipropylphthalate | 0.756981478997596 |
| Biochanin-7-O-glucoside | 3.48334321997786 | Dexamethasone | 1.42378033244877 | Glyceryl linolenate | 0.696691200862293 |
| Pyrogallol | 2.20727896142297 | 2-Acetylfuran | 1.73951241847996 | Indole-3-carboxylic acid | 0.492099418489184 |
| Biochanin A | 2.02839864409827 | Yuccoside C | 8.93749338390021 | Isoleucine | 1.75547607986049 |
| Licoricesaponin G2 | 1.0830182175457 | Cholic acid | 0.0932629104812101 | Isomyristicin | 4.24258648019107 |
| Embelin | 1.25593505496837 | 8-hydroxy-5,7-dimethoxy-2-phenyl-2,3-dihydrochromen-4-one | 1.73443626304946 | Kainic acid | 0.835337287375732 |
| Licoricesaponin H2 | 0.077312357869048 | (S)-Nerolidol 3-O -[a-L-Rhamnopyranosyl-(1->4)-a-L-rhamno | 4.36596518985095 | Linalyl anthranilate | 4.35443449040731 |
| Methyl hexadecanoate | 1.67359081445232 | Thalictroidine | 3.20008759067462 | Loliolide | 1.76072749731025 |
| Biorobin | 1.3805720179548 | Zizyberanalic acid | 5.19211192774986 | Lucidone C | 0.66165608942936 |
| 7,8-Dihydroxycoumarin | 0.0705040425884708 | Curcumenol | 2.98396872036888 | m-Xylene | 3.07023948806873 |
| 1,7-bis(4-hydroxyphenyl)heptane-3,5-diol | 8.65281434810783 | Ganoderiol F | 5.87050874533454 | Maltol | 1.48356379229101 |
| P-Anisic acid | 1.56836798522112 | Polyporusterone A | 0.497537456851945 | Matricin | 2.11967578266994 |
| Benzene-1,2,4-triol | 2.84149699686803 | 3-Methylbenzaldehyde | 4.29092179310468 | Melatonine | 2.4599001464385 |
| Ginsenoside Ro | 7.96964974830921 | Quillaic acid 3-[galactosyl-(1->2)-glucuronide] | 7.13583825549019 | Methyl cinnamate | 2.376068577926 |
| 2,3-bis[(4-hydroxy-3-methoxyphenyl)methyl]butane-1,4-diol | 1.70626880648654 | Nerolidol | 3.98945754576623 | N-(14-Methylhexadecanoyl)pyrrolidine | 0.764265962186789 |
| 5-OXO-D-PROLINE | 1.90544894868019 | 1a,5,7a-Trimethyl-2,2a,6,6a,7a,8,9,9a-octahydrobisoxireno[4,5:8,9]cyclodeca[1,2-b]furan-4(1aH)-one | 1.94540155336008 | Napelline | 28.4382005078822 |
| Fumaric acid | 5.95145351500552 | Leonurine | 0.407811988042693 | Nicotinamide | 90.643591916949 |
| SUCCINATE | 2.33468573605285 | Genistein | 1.07113372745436 | Norharman | 2.06215845532571 |
| Asiaticoside | 2.36111170783189 | 6-Methoxymellein | 3.15388233344996 | p-Hydroxybenzaldehyde | 0.627785243363037 |
| Paracetamol | 0.60454108566209 | Proline | 4.75928831569701 | Pesticide3_Propoxur_C11H15NO3_Baygon | 3.3223985147109 |
| Vicenin 2 | 5.58909334482582 | Sugiol | 2.89993065135799 | Phenethylacetate | 0.308138618630595 |
| Chikusetsusaponin IV | 0.560770207436203 | Harmaline | 49.1022506625474 | Phenylalanine | 0.958552047657215 |
| Salicylic acid | 2.35508320449978 | Dehydrodiisoeugenol | 2.34627725664902 | Physcion | 0.715937534777993 |
| Benzoic acid + 1O, 1MeO, O-Hex | 1.39928701426102 | 2-Hydroxy-4-methoxybenzaldehyde | 1.30167479887442 | Progesterone | 2.10726732003544 |
| 5,9-dihydroxy-5,7,7-trimethyl-4,5a,6,8,8a,9-hexahydro-1H-azuleno[5,6-c]furan-3-one | 0.177246336211291 | 3',4'-Dihydroxyacetophenone | 1.03560598772652 | Suberosin | 3.71657049811725 |
| Genistein | 0.737511235551429 | Nicotinic acid | 1.59708904950445 | Theaspirone A | 9.41337717947547 |
| kojic acid | 4.47691781685327 | 3-Hydroxystigmast-5-en-7-one | 2.73288222120802 | Venoterpine | 2.13039950291741 |
| Soyasapogenol B base + O-HexA+HexA+dHex | 0.891567578181363 | Furanofukinin | 1.72094637294839 | 2-Methylbenzaldehyde | 3.99727833718911 |
| Phenylalanine | 3.52236252819933 | 2,6-Dimethyl-1,4-benzenediol | 0.839971151379788 | Naphthalene-1,2-diol | 3.42024102920198 |
| D-Gluconic acid | 3.55074946340079 | Schisandrin B | 4.82392654710121 | 2-Nonanone | 5.68944168380054 |
| adipic acid | 4.94237473853012 | Methyl 4-hydroxycinnamate | 0.0342840944456261 | Isoliquiritigenin | 0.91707302451936 |
| Pseudojervine | 9.97679545291362 | Ethyl p-anisate | 0.712650512264988 | trans-4-Coumaric acid | 0.581769894314271 |
| Vanillic acid | 2.30037693842972 | Pogostone | 4.95687765532473 | 8-Epidiosbulbin E acetate | 0.607395391944951 |
| Quercetin | 7.08984882182055 | Propranolol | 2.28264579641115 | Paracetamol | 1.85825362584217 |
| Spirostane -2H, + 1O, O-Hex-dHex, C6H9O4 | 5.55320809401544 | Sesamol | 3.34956679735703 | Daidzein | 4.33114661189396 |
| Methylparaben | 0.107459300472321 | 3-Butylidenephthalide | 2.38618937271422 | Formononetin | 1.31490129086891 |
| Methyl Heptadecanoic acid | 1.29647510938376 | 2-(3,7-Dimethyl-2,6-octadienyl)-4-hydroxy-6-methoxyacetophenone | 0.228629463261941 | Tectochrysin | 3.30597051047134 |
| Ethyl myristate | 0.336283060627253 | Berberine | 3.87590447673611 | Paeonol | 0.53556202241085 |
| 4-Methoxysalicylic acid | 3.40210097174116 | Ginsenoyne B | 3.55992774166103 | Fraxinol | 4.94714933615087 |
| Kynurenic acid | 0.455750413272073 | alpha-Hydrojuglone 4-O-b-D-glucoside | 23.446095299651 | 2,4-Dimethylbenzaldehyde | 0.782457345969607 |
| Aconitic Acid | 0.826108659393605 | (S)-Pterosin P | 3.256806466176 | 3-Furfuryl 2-pyrrolecarboxylate | 0.0938136479901751 |
| DL-beta-Hydroxybutyric acid | 0.676680559189181 | BIOTIN | 2.20996161987053 | Homoeriodictyol | 2.54669544952734 |
| Madecassoside | 10.3047881211385 | Methyl 2-furoate | 3.81636514196318 | 2-Cyclohexen-1-one, 4-hydroxy-4-(3-hydroxybutyl)-3,5,5-trimethyl- | 1.62190615867741 |
| Methyl-4-hydroxy-3-methoxybenzoate | 2.39963393232483 | (3beta,5alpha,6beta,9alpha,22E,24R)-5,9-Epidioxyergosta-7,22-diene-3,6-diol | 1.98597778822078 | Quercetin | 0.625831934265166 |
| Linoelaidic acid | 0.697019633530305 | 4-Ethyl-2-methoxyphenol | 4.05490172611963 | Tuberostemonine | 26.564961982267 |
| Gambogenic acid | 1.86348388953408 | 1alpha-Hydroxyarbusculin A | 1.8705132215064 | (E/Z)-cinnamic acid | 2.16954329759974 |
| 8-Desoxygartanin | 0.924945652118666 | Phenacetin | 2.93529888926538 | Bufalin | 3.16240525246127 |
| Fraxinol | 2.44573227989922 | Azukisaponin IV | 4.88609974188023 | Glycitein | 3.77092923196813 |
| Sebacic acid | 0.00145276041334551 | Withanolide A | 4.09045340241316 | Genkwanin | 2.93164080147809 |
| 1-Naphthalenecarboxylic acid, 5-[2-(2,5-dihydro-2-oxo-3-furanyl)ethyl]decahydro-1,4a-dimethyl-6-methylene- | 1.93090828239364 | 3,12-dihydroxy-4,6a,6b,11,12,14b-hexamethyl-1,2,3,4a,5,6,7,8,9,10,11,12a,14,14a-tetradecahydropicene-4,8a-dicarboxylic acid | 2.71651268851197 | Naringenin chalcone | 2.85350383898932 |
| Azelaic acid | 1.65827488674926 | 1-Hydroxyepiacorone | 0.0314549737461948 | Protolichesterinic acid | 0.156935125252345 |
| Santalyl acetate | 3.47156346685598 | Gentiatibetine | 0.0881884125869631 | Tyrosine | 3.23509134686536 |
| Dibutylphthalate | 0.86804341013553 | Prunetin | 2.97189465924524 | 2-METHYLMALEATE | 1.13857902893337 |
| Lotaustralin | 2.93233514019684 | Germacrone | 1.44079324635966 | VERATRIC ACID | 2.68070252380294 |
| 7-Hydroxy-1,7-bis(4-hydroxy-3-methoxyphenyl)-1-heptene-3,5-dione | 0.379873447993543 | alpha-Cyperone | 1.16142217758407 | 4-hydroxyphenylacetic acid | 1.19972193723032 |
| O-Methylcorypalline | 2.65873290381819 | Coumaperine | 2.94833641802727 | columbianetin | 2.8019641420018 |
| gamma-Eudesmol rhamnoside | 1.5307951412794 | (R)-8-Acetoxycarvotanacetone | 3.69360030020158 | 7-hydroxy-1,4a-dimethyl-9-oxo-7-propan-2-yl-2,3,4,4b,5,6,10,10a-octahydrophenanthrene-1-carboxylic acid | 0.74948183344888 |
| trans-pterostilbene | 2.80723812390791 | 2,3-dihydroxypropyl hexadecanoate | 1.6003459792979 | Piperonylic Acid | 0.342535536100877 |
| Phenylpropanolamine | 1.54094065482382 | 28-[Glucosyl-(1->6)-glucosyl]oleanolic acid 3-arabinoside | 0.802428878101089 | 9-hydroxy-1,4a-dimethyl-7-propan-2-yl-2,3,4,9,10,10a-hexahydrophenanthrene-1-carboxylic acid | 3.93866326251934 |
| 7-methoxy-9,10-dihydrophenanthrene-2,5-diol | 0.563578548217949 | 2-Hydroxyadenine | 14.0431014386047 | PYRIDOXINE | 1.67172179914024 |
| Benzenepropanamide, N-[2-(acetyloxy)-1-(phenylmethyl)ethyl]-alpha-(benzoyl | 2.54030751586784 | Dihydro-5-pentyl-2(3H)-furanone | 0.691497474283665 | Lucidenic acid D1 | 3.77984917503977 |
| 3,4-Dihydrocadalene | 3.19381799342138 | Ginsenoside F3 | 4.96960378790626 | Nobiletin | 2.77550296301166 |
| Isosinensetin | 6.07715614385654 | 12-Ketoporrigenin | 1.69095891420433 | 4,4,8,10,14-pentamethyl-17-(4,5,6-trihydroxy-6-methylheptan-2-yl)-2,5,6,7,9,15-hexahydro-1H-cyclopenta[a]phenanthrene-3,16-dione | 11.4799542617188 |
| (4,7,7-trimethyl-3-bicyclo[2.2.1]heptanyl) (E)-3-(4-hydroxy-3-methoxyphenyl)prop-2-enoate | 1.93618498805063 | Jatrorrhizine | 2.74420031815666 | Vulgarole | 1.45253121910181 |
| 2-Hydroxyacetophenone | 3.47498809216064 | Parthenolide | 0.2757232008896 | Gibberellin A17 | 1.03249465611356 |
| 6beta-Hydroxy-3-oxo-12-oleanen-28-oic acid | 13.0003424822359 | Kaempferide | 2.96323608605428 | Linoleic acid | 4.99409967921499 |
| 3,8-dihydroxy-3,8-dimethyl-5-propan-2-ylidene-1,2,3a,4,7,8a-hexahydroazulen-6-one | 3.93770966378696 | 2,4,6-trihydroxy-5-[1-(4-hydroxy-1,1,4,7-tetramethyl-1a,2,3,4a,5,6,7a,7b-octahydrocyclopropa[h]azulen-7-yl)-3-methylbutyl]benzene-1,3-dicarbaldehyde | 0.684584692202942 | Curcolone | 1.13518372585241 |
| Sinapoyl aldehyde | 3.20103267468101 | 2-Phenylethyl 3-methylbutanoate | 2.66972807682216 | Persin+A643A601:A659 | 0.175062292984676 |
| 2-Methoxy-4-methylphenol | 0.14379292247816 | 1,7-bis(4-hydroxyphenyl)heptan-3-one | 0.0610432177655609 | Oleamide | 1.05563349941945 |
| Zanthodioline | 0.22517680007183 | 7-methoxy-6-(1,2,3-trihydroxy-3-methylbutyl)chromen-2-one | 2.52354669102199 | Camelledionol | 2.46178808365853 |
| Sarracenin | 49.1278890079002 | 28-Galloylglucosylpomolate 3-arabinoside | 1.65296057813452 | Isopeonol | 0.415201429358347 |
| Dioctylphthalate | 2.60327505974871 | Isosativan | 0.737329592053405 | Grandisin | 3.50259673096245 |
| (4S,5S,10R)-4-Hydroxy-5-methoxy-10-methyl-2,7-oxecanedione | 0.26443552162497 | 3-n-Butylphathlide | 1.98883575125286 | Phthalic anhydride | 0.659837220647923 |
| Sideridiol | 0.918994411763847 | Di-2-furanylmethane | 3.52038582508973 | Pyroglutamic acid (not validated, isomer of 88) | 1.83760416886141 |
| Cyclopassifloside III | 5.79813448725137 | Withanolide B | 18.9974862136239 | Tsugaric acid B | 0.470520250553118 |
| Solasodine | 3.07999083969037 | Hederacoside C | 5.92337515913916 | Soyasapogenol E base + O-HexA-Hex-dHex | 12.9565211870545 |
| Junosmarin | 2.79424388924236 | Lucidenic acid M | 0.700201508241976 | Palmatine | 1.14150178580317 |
| Tangeritin | 2.38209875226258 | Myristicin | 4.04096086196327 | Pyrrolo[1,2-a]pyrazine-1,4-dione, hexahydro-3-(1-methylethyl)- | 1.15628317369314 |
| Egonol glucoside | 2.46754741434266 | Reichsteins substance S | 2.519565374326 | Cinnamic acid | 3.52609269953978 |
| Maltoxazine | 2.87057039623428 | Pimara-9(11),15-dien-18-oic acid | 2.07142645277989 | 2-Hexyl-5-[2-(4-hydroxy-3-methoxyphenyl)ethyl]furan | 1.8262124921029 |
| Hesperetin 5-O-glucoside | 7.45723289891715 | (4Z,7Z)-5,9,9-Trimethyl-11-oxabicyclo[8.2.1]trideca-1(13),4,7-triene-6,12-dione | 2.35797426865107 | Biochanin_A | 2.44213521375146 |
| 3-(4-Methoxyphenyl)-2-propen-1-ol | 2.47616354162436 | Guttiferone A | 9.76174712441124 | isoimperatorin | 1.33487783546261 |
| 6,7,8-trimethoxychromen-2-one | 2.44997699459414 | Isomasticadienonic acid | 4.18999341841232 | Methyl nicotinate | 1.1564449793486 |
| Santonin | 1.02025314301001 | Ixocarpalactone B | 5.3468358550069 | 2,6-di-tert-Butylphenol | 1.36550471243376 |
| Polyporusterone E | 18.476059018059 | Azuleno[5,6-c]furan-1(3H)-one, 4,4a,5,6,7,7a,8,9-octahydro-4,8-dihydroxy-6,6,8-trimethyl- | 0.186362622415891 | 2-[1-[1-hydroxy-10,13-dimethyl-3-[3,4,5-trihydroxy-6-[[3,4,5-trihydroxy-6-(hydroxymethyl)oxan-2-yl]oxymethyl]oxan-2-yl]oxy-2,3,4,7,8,9,11,12,14,15,16,17-dodecahydro-1H-cyclopenta[a]phenanthren-17-yl]ethyl]-4,5-dimethyl-2,3-dihydropyran-6-one | 4.37455919153407 |
| Pectolinarin | 2.75934196895298 | Dubinidine | 0.644116203535125 | 5-[2-(3-Furyl)ethyl]-8a-(hydroxymethyl)-5,6-dimethyl-3,4,4a,5,6,7,8,8a-octahydro-1-naphthalenecarboxylic acid | 0.879425279313182 |
| Polyporusterone D | 2.01257271867303 | Methylisoeugenol | 2.17174159025942 | (3xi,6xi)-Cyclo(alanylvalyl) | 3.27696654540081 |
| Cuelure | 0.354776142755055 | Tetramethylpyrazine | 0.885925535412434 | Acevaltrate | 14.3512333402797 |
| Dihydrocapsaicin | 0.962754257232952 | 7-(2-hydroxypropan-2-yl)-1,4a-dimethyl-2,3,4,5,6,7,8,8a-octahydronaphthalen-1-ol | 0.455055270281471 | Gingerenone A | 0.815713998026102 |
| Xanthyletin | 1.55336051124926 | Curdione | 0.786468763859783 | (5x,6x)-5,6-Epoxyergosta-7,22-dien-3-ol | 1.31440118127473 |
| 6-Demethoxytangeretin | 2.30191744859254 | Meperidine | 0.478019219959504 | Lucidumol A | 8.27157962449928 |
| 5-O-Methylembelin | 1.40957596678758 | Ixocarpalactone A | 5.78887860503836 | Beauverolide I | 3.90915913094999 |
| Dihydrovaltrate | 0.0567397779164447 | Curzerenone | 3.68886369008541 | 6-[3-[(3,4-dimethoxyphenyl)methyl]-4-methoxy-2-(methoxymethyl)butyl]-4-methoxy-1,3-benzodioxole | 1.34676286586717 |
| Medicagenic acid | 0.779942553718349 | Abscisic acid | 2.13212535960391 | Ixocarpanolide | 4.38452373666484 |
| Armexifolin | 1.25288973058119 | Methyl lucidenate F | 5.53249591832817 | Genipic acid | 0.650801235122574 |
| Sophoraflavanone B | 0.821658763496434 | (14alpha,17beta,20S,22R)-14,20-Epoxy-17-hydroxy-1-oxowitha-3,5,24-trienolide | 2.30345795166586 | N-cis-Feruloyltyramine | 0.658010116595875 |
| D-beta-homophenylalanine-HCl | 3.22507175275987 | 2-Phenylethyl beta-D-glucopyranoside | 2.39710932647949 | 3-Formylindole | 1.50155748532995 |
| 4-[2-(2,6-dimethoxy-4-prop-2-enylphenoxy)-1-hydroxypropyl]-2-methoxyphenol | 31.349237302156 | Sissotrine | 6.00293339142862 | Arctigenin | 1.5162704358139 |
| Polyporusterone F | 0.166259400036113 | Dihydroactinidiolide | 1.05645360668031 | Adenosine | 4.91406790706271 |
| 2'-Hydroxyacetophenone | 3.92649846343627 | 2,2,6b,9,9,12a-hexamethyl-4a-[3,4,5-trihydroxy-6-(hydroxymethyl)oxan-2-yl]oxycarbonyl-10-(3,4,5-trihydroxy-6-methyloxan-2-yl)oxy-1,3,4,5,6,6a,7,8,8a,10,11,12,13,14b-tetradecahydropicene-6a-carboxylic acid | 3.5800601098773 | Dihydrodioscorine | 0.606856642743286 |
| Tanshinone IIA | 2.82392184715478 | Scopoletin | 0.487534002331138 | 4-Androstene-3,17-dione | 1.28143292691482 |
| L-2,3-DIAMINOPROPIONIC ACID | 4.18959330205254 | ISOPALMITIC ACID | 2.06999548580761 | alpha-Asarone | 0.84546249447081 |
| Isoelemicin | 0.618057576052945 | SPERMIDINE | 0.0348858537983939 | Azelaic acid | 0.775475050393366 |
| Gambogenic acid | 5.34523696568103 | Barogenin | 1.66636559678339 | 3,9-Dimethoxypterocarpan | 4.40543151972689 |
| Gingerenone B | 0.25340011138013 | Austricine | 2.11271609428653 | P-Anisic acid | 1.82734502142128 |
| 3-(3-methylbut-2-enyl)purin-6-amine | 0.328674731855628 | Loureirin A | 0.953078194803678 | 2,2,6,6-Tetramethyl-4 piperidone | 0.447132323919828 |
| 11,17,21-Trihydroxypregn-4-ene-3,20-dione | 3.27135187696215 | 2,3-Secoporrigenin | 0.843087413690172 | beta-Bourbonene | 5.43705395924101 |
| Thermophillin | 0.84344705538715 | Karakoline | 4.07152420892333 | Floribundine | 2.82107606484442 |
| Epoxyganoderiol C | 1.60431963590794 | Kaempferitrin | 3.16258398752648 | Pterosin O | 1.00331276593045 |
| blennin C | 2.78033467867229 | Withaphysacarpin | 2.20831379945135 | Isotalatizidine | 4.21303100527824 |
| 3,5,7,8-tetramethoxy-2-(3,4,5-trimethoxyphenyl)chromen-4-one | 2.72591159992015 | Tyramine | 3.61416978316648 | Nordihydrocapsaicin | 3.61338027109588 |
| (-)-5,7-Dihydroxy-3-(4-hydroxybenzyl)-4-chromanone | 2.65864262756018 | 4,5-Dihydropiperlonguminine | 3.33282453119708 | Methoxyeugenol | 2.1522874676116 |
| 6,8-dihydroxy-2,2,4,4-tetramethyl-7-(3-methylbutanoyl)-9-(2-methylpropyl)-9H-xanthene-1,3-dione | 2.70721591262864 | Methyl vanillate | 1.82707620767835 | Kojic Acid | 3.78567871866536 |
| 6-Hydroxyshogaol | 1.88221533427773 | beta-Elemonic acid | 1.13730871657347 | Santin | 1.4249451768342 |
| Piperdial | 1.70844509528381 | Coumaric acid | 0.557070133003572 | Corosolic acid | 3.87375360398571 |
| Schisandrin | 4.5164990449346 | (+/-)-Jasmonic acid | 4.047699822045 | Bisacurone epoxide | 4.02794501409669 |
| (5alpha,6beta,14alpha,20R,22R)-5,6,14,20,27-Pentahydroxy-1-oxowith-24-enolide | 3.86905524977938 | 4-Hydroxybenzoate | 0.335233291425268 | trans-Grandmarin | 0.670758874564011 |
| Pterosin E | 3.75358951549677 | 3-(2-Hydroxy-3,4-dimethoxyphenyl)-7-chromanol | 0.960371174623215 | 1,4,5-Naphthalenetriol | 2.42435728279236 |
| Auxin b | 2.58991201631805 | Polyporusterone B | 0.988915321069711 | Coniferyl aldehyde | 0.573145065229029 |
| Valerophenone | 3.85317412873841 | Procurcumadiol | 2.47444346789192 | Erinacine C | 4.73865665818198 |
| (6alpha,22E)-6-Hydroxy-4,7,22-ergostatrien-3-one | 0.198426213331384 | Isofraxidin | 5.30910773695524 | Methoxycinnamic acid | 0.473221856920357 |
| (2Z,6E,10E)-12-hydroxy-6,10-dimethyl-2-(4-methylpent-3-enyl)dodeca-2,6,10-trienoic acid | 1.27638938840772 | Talatisamine | 30.3290196469691 | 3-(1,1-Dimethylallyl)herniarin | 1.91228925920366 |
| Eugenitin | 1.32736473438311 | Oleanane -4H, +2O, 1COOH, O-HexA, HexA | 1.72454741295336 | alpha-Linolenic acid | 1.82502391581638 |
| 17-Hydroxy-15,16-epoxykauran-18-oic acid | 1.31410703917765 | (4beta,5beta,6beta,14beta,15alpha,20S,22R)-5,6-Epoxy-4,14,15-trihydroxy-1-oxowitha-2,24-dienolide | 2.93950760841641 | Phytolaccasaponin B | 5.98148764969216 |
| Higenamine | 0.781823157951732 | Furohyperforin | 2.07323474508165 | Isoglabrolide | 2.21654065957744 |
| Ambonic acid | 0.276835361908389 | Ginkgolic acid (C13:0) | 1.0320084582809 | Nerolidyl acetate | 0.274846713696267 |
| alpinetin | 0.870404469174417 | 5-O-Demethylnobiletin | 2.69626035551351 | Sinensetin | 0.48462335553719 |
| Chinenoside VI | 2.77258908566783 | Geranial | 1.17669956330089 | Sebiferic acid | 6.52598053202919 |
| 5'-(furan-3-yl)-4a-hydroxy-4,7-dimethylspiro[5,6,7,8a-tetrahydro-1H-naphthalene-8,3'-oxolane]-2,2'-dione | 4.16436753741563 | Panaquinquecol 1 | 1.70562086345308 | 3,19-Dihydroxyurs-12-ene-23,28-dioic acid | 0.256732121166347 |
| 3,7,11,15,23-Pentaoxolanost-8-en-26-oic acid | 1.96985034751117 | Dehydronuciferin | 2.1108190511513 | Ganoderic acid beta | 17.2298716456373 |
| alpha-Irone | 1.00660757109903 | Ginsenoyne D | 4.33733294870952 | Kaempferol | 4.44183550436684 |
| 5-Isopropyl-2-methylphenol acetate | 0.172217879131153 | Avenacoside A | 2.14906121250563 | Hexadecanedioic acid | 0.501697254693214 |
| Polyporusterone C | 1.77588768459818 | Linalyl propionate | 0.236100749670023 | Hydroxyferulic acid | 0.638381644254346 |
| Glutaric acid | 0.430421706325541 | Pimelic acid | 1.83209131478147 |  |  |

| **Table S2: DN and JCYSTLF-related targets** |
| --- |
| **1141 DN-related targets** |
| HTR2A, ACE, ADRA2C, AOC3, CCR2, CCR5, DRD2, EDNRA, FCGR2A, IGF1R, INSR, MAP3K5, NR3C2, MAPK12, MMP1, MME, ROCK1, SLC5A2, TGFBR1, UTS2R, YWHAZ, ACCS, TUG1, LRG1, ADORA1, PRKAB1, PRKAA1, PRKAA2, ABCA1, ABCC8, ACACB, SLC33A1, ACE2, ACKR2, ACOX1, ACR, ACSS2, ACTA2, ACTB, ACTG2, ACTN4, ACVRL1, ACVR1, ADAM10, ADAM17, ADCY8, ADD1, ADD2, ADH1B, ADIPOQ, ADM, MLLT3, AFM, ADGRG1, AGTR1, AGTR2, AHSA1, AIFM1, AIMP2, AKR1A1, AKAP1, AKT1, ALB, ALDH2, AKR1B1, ALKBH1, ALPK1, ENPEP, LRPAP1, NPPA, NPPB, ANG, ANGPTL2, ANGPTL4, ANGPT1, ANGPT2, AGT, ANKRD1, PRMT1, ANO1, APLN, APEX1, APLNR, LPA, APOA1, APOA4, APOA5, APOB, APOC1, APOC3, APOE, APOH, APOL1, APOL3, APOM, APRT, AQP1, AQP11, AQP2, AQP5, ARAP1, GRK2, GRK3, CDKN2A, ARF6, ARG1, ARG2, ARID2, ARRB1, ARRB2, NAT2, PYCARD, SMPDL3B, ATP2A2, ATF6, ATM, ATN1, ATP5F1B, ADAMTS13, AXIN2, AZIN1, B2M, B3GALNT1, TNFAIP1, BSG, BASP1, BCL2, BCR, BDNF, BECN1, BEST1, TGFBI, HSPA5, BDKRB2, BMP2, BMP4, BMP7, BNIP3, BBOX1, BRD2, BRD4, CYP11B2, CD163, C3AR1, C5AR1, CDH13, CDH2, CADM1, CALB1, CALD1, CALCRL, CAPN10, AZU1, CASC2, CASP3, CASP7, CASP8, CASP9, CAT, CTSB, CTSD, CAV1, CPB2, CCHCR1, CCL1, CCL2, CCL20, CCL5, CCN2, CCNE1, CCNG2, CCR1, CCR6, ALCAM, CD2AP, CD36, CD38, CD40LG, CD44, CD59, CD5L, CD68, CD80, CDAN1, CDC42, CDK5, CDKAL1, CDKN1A, CDKN1B, CEBPB, CP, CETP, LINC00472, CFD, UGT8, CHI3L1, CHN2, CHIT1, CHPT1, TRIP10, LINC01619, CLU, CMKLR1, CNBP, CNDP1, CNDP2, CNKSR3, CNR1, COL1A1, COL1A2, C3, COL4A1, COL4A5, C5, COL6A3, COL8A1, COL8A2, COL11A2, CFL1, COL18A1, COPB2, CORO2B, COX8A, CYP19A1, CYP24A1, CYP27B1, CYP2R1, CYP4B1, CPT1A, CPT1B, CPT2, CR1, ORAI1, CREB1, CREM, CRISP2, CRK, CRP, CRTC1, CSF1R, CSNK2A2, VCAN, CTNNB1, SLC7A2, CUBN, CUL5, GJA1, GJB1, CXCL6, PPBP, CXCL9, CXCR1, CXCR2, CXCR4, CXCL10, CXCL16, CYBA, CYBB, CYLD, CST3, CST2, DAAM1, DAPK2, DBP, DCTN4, DDAH1, DDAH2, DDIT3, DECR1, DEFA1, DEFA3, DENR, DERL2, DES, DGKA, DGKH, HSD11B2, SORD, DKK1, DMP1, DMTF1, DNM1L, DNMT3A, DNMT3B, DNMT1, DDT, HLA-DPA1, DPP4, DPYD, CRMP1, DPYS, HLA-DQA1, HLA-DRB1, DRD3, DRG1, DSEL, DSPP, DUOX1, DUOX2, DUSP1, DUSP26, DUSP4, EIF2AK3, E2F1, E2F3, EDA, EDN1, EFHD2, EFNA1, EGF, EGFR, ENG, EGR1, EHMT1, EIF3M, ELAVL1, ELAVL2, ELF3, ELMO1, ENO2, ENOX1, ENPP1, ENTPD1, EP300, EPAS1, EPHA1, EPHB2, EPO, ERBB4, EREG, ETF1, ERP44, ERRFI1, ESAM, ESCO1, ESM1, ESR1, ETS1, EZH2, EZR, F10, F5, FABP4, FABP2, FABP1, FAR2, PTK2, PTK2B, FASN, BTRC, FBXW7, AHSG, FFAR2, FGF1, FGF11, FGF13, FGF2, FGF21, FGF23, FHL2, FN1, FIS1, FITM1, FN3K, FNDC5, FOXA1, FOXC1, FOXO1, FOXP1, FOXP3, FPR2, FRMD3, FSCN1, FSD1, FSD1L, FST, FTO, FUT8, G6PD, GABPA, GLIPR2, GAS6, GAST, AMT, GUCY1A1, GDE1, GDF15, GFPT1, GFPT2, GHR, GHRL, GIPR, GLP1R, PRKCSH, GCG, SLC2A4, GORASP1, GPR158, GPBAR1, GPC5, GPRC5B, GPX1, GPX3, GPX4, GRAP, GRAP2, GRB2, GREM1, GREM2, GRK4, GRK6, GRN, GSDMD, GSR, GSK3B, GSTM1, GSTM2, GSTP1, GSTT1, SLC2A1, SLC2A12, SLC2A2, H2BS1, HAVCR1, HAVCR2, HDAC2, HDAC4, HDAC5, HEBP1, HES1, HFE, CD74, HGF, HHIP, HIF1A, HLA-B, HMCN1, HMGA2, HMGB1, HMGN1, HMOX1, HNF1A, HNF1B, HNF4A, HNMT, HPGDS, HPSE, HP, HSPA1A, HSPA1B, HSPA4, HSPB1, HSPB2, HSPB3, GCK, HAS2, EPHX2, HYOU1, IL12RB1, IAPP, IGFBP1, IGFBP3, IGFBP4, IGFBP7, ICAM1, IPCEF1, ID2, IDUA, IGF2BP2, IFNG, IGF1, IGF2, NFKBIA, IKBKB, IL10, IL13, IL15, IL17A, IL17B, IL18, IL18R1, IL19, IL1A, IL1B, IL1R1, IL1RN, IL2, IL20, IL22, EBI3, IL33, IL34, IL4, IL5, IL6, IL6R, IL6ST, CXCL8, IL1RL1, KPNA2, INHBC, ISYNA1, INS, INTU, IPPK, IQGAP1, IRAK1, IRAK4, IRS1, IRS2, SPINK1, ITGA1, ITGA2B, ITGAM, ITGB3, ITPR1, JAG1, JAK1, JAK2, JUN, KRT16, KCNJ11, KCNH7, KCNQ1, KDM6A, KEAP1, KHDRBS1, KHK, KIRREL1, CAMKK2, KLF15, KLF2, KLF4, KLF6, KLK1, KL, KMT2A, KNG1, PRKCA, PRKCB, PRKCE, PKM, SYK, LAD1, LAMB2, LAMP2, LARGE1, LCK, LCN1, LGALS1, LGALS3, LEP, LEPR, GLO1, LIMK2, LIPC, LPL, LMNA, LIN28A, ALOX12, LOXL2, LPAR1, LPAR3, LRP2, LRP5, LRP6, LRRC7, LTBP1, SELL, SELP, MTDH, LYZ, MAP3K1, MAP3K7, MAK16, MALT1, MAP6, MARK2, MASP2, MBL2, MBNL2, MCM3, MDM2, MEFV, MEP1B, MCF2L2, MFAP1, MFHAS1, MFN2, MGAM, MGP, MIF, OGN, MINDY4, MIOX, MIXL1, MDK, MAPK1, MAPK3, MAPK8, MAPK14, MAP1LC3A, MLXIPL, MMP10, MMP12, MMP2, MMP3, MMP7, MMP8, MMP9, MMRN1, MSN, MOK, SLC16A3, MLN, MAP2K2, MAP2K7, MPRIP, MARCKSL1, ABCC6, ABCC11, MRTFA, MSX2, MTHFS, MTHFR, MTOR, MCAM, MSC, MYOCD, MYCN, MYD88, MB, MYH2, MYH9, MZF1, NAV3, NBL1, NCALD, NCK1, NDUFS3, PCSK1, NDN, NTN1, AVP, NFE2L2, NFAT5, NFIA, NFIB, NFIC, NFIX, NFKB1, LCN2, NLRC4, NLRC5, NLRP1, NLRP3, NLRX1, NMUR2, NOD2, NOS1, NOS2, NOS3, NOTCH1, NOTCH2, NOTCH3, NOX1, NOX3, NOX4, NOX5, NPHS1, NPY, NQO1, NR0B2, NR1H3, NR1H4, NR1I2, NR2C2, NR4A1, NRG4, NRK, NSA2, NTRK1, NTRK2, NUAK1, NCL, NUP62, OR10A4, OGA, OLR1, OPTN, DDOST, BGLAP, SPP1, P2RX7, PIK3C2B, TP53, PIK3R1, PIK3R2, PLA2G1B, PACSIN2, PAEP, PLA2G7, PLA2G15, SERPINE1, SERPINB2, PALD1, PALLD, ADIPOR1, ADIPOR2, PAQR3, F2R, F2RL1, PARN, PARP1, PAX2, PDE3A, PDE5A, PDGFA, POLDIP2, PDK1, PDPN, PEA15, PEBP1, PECAM1, SERPINF1, PGC, MPO, PES1, HSPG2, PDGFRA, PDGFRB, PTGS1, PTGS2, BGN, DCN, PHLPP1, PIAS4, PICK1, PIN1, PITX2, PIK3CA, PIK3CB, PIK3CD, PIK3CG, PKD1, PLEKHH2, PLEKHO1, PLA2R1, PLB1, PLIN2, PLK2, PLG, PNPLA2, PNO1, POU2F3, NPHS2, PON1, PON2, POSTN, PPP1R8, PPARA, PPARD, PPARG, PPIA, PPIG, PPARGC1A, PROC, PROS1, PRSS55, PSMA6, PSMD9, PTAFR, PTBP1, PTEN, PTGDS, PTHLH, PTH, PTPN11, PTPN2, PTPN6, PTPA, PTX3, CRYZ, RAB38, RAB3A, RABGEF1, RAC1, RDX, AGER, GPRC5A, RAMP2, RAP1A, RARRES2, MRAS, RB1CC1, RBM45, RBMS3, RCAN1, RCBTB1, REM1, RENBP, REN, ATP6AP2, RBP4, RETN, RFC1, RHBDF2, RIPK2, RIPK3, RPL36A, RPLP0, RMC1, RMDN1, RMDN2, RMDN3, RNF185, RNF19A, RNASE2, ROCK2, ROS1, RAPGEF5, RRAS, RPS19, RPS27A, RPS6, SNRNP70, S100B, S100A4, S100A8, S100A9, SLC12A1, SLC12A3, SLC17A5, SLC22A2, SLC22A3, SLC22A6, SLC22A8, SLC52A1, SLC52A2, SAA1, SAA2, SASH1, SELENBP1, SLC5A1, SCAF4, SCAF8, SCD, SDC2, CXCL12, SEMA3A, SEMA6A, SEPTIN7, SETD7, SFI1, SGK1, SHBG, INPPL1, ST3GAL4, SIRT1, SIRT3, SIRT4, SIRT6, SIRT7, SKIL, SKP2, SLC9A8, SLBP, SLIT2, SLPI, SMAD1, SMAD2, SMAD3, SMAD5, SMAD6, SMAD7, SMG1, SMURF2, SNAI1, SLCO6A1, SOAT1, SOCS1, SOCS2, SOCS3, SOCS5, SOD1, SOD3, SOD2, GH1, SOS1, SOSTDC1, SOST, SOX2, SOX6, SP1, SPHK1, SPARC, SPZ1, SQSTM1, SREBF1, SREBF2, SORBS1, SRGAP2, SRRM2, SRXN1, SRY, STAT5A, STAT5B, STAM2, STAP2, STAT1, STAT3, STC1, NR5A1, STIM1, STIM2, STK11, STS, RBPJ, SI, SUV39H1, SYBU, CARS1, SYT1, SYVN1, WARS1, TSC22D1, TAS2R13, TALDO1, TBC1D31, TBX1, TCF7, CCT2, TERF2IP, TERF1, TET2, GTF2H1, RELA, TCF7L2, TFG, TNFAIP8, TFPI, TFRC, TGFA, TGFB1, TGFB2, TGFB3, TGFBR2, TGIF1, TGM2, THG1L, TXN, TIMM44, TIMP1, TIMP2, TIMP3, TINAG, TKT, TLR2, TLR4, TNFSF10, TNF, LTA, TNMD, TNFRSF1A, TNFRSF1B, FAS, TNS2, TPD52, TNFRSF10D, TNFRSF11B, TRAF5, TRAF6, THBD, TRDN, TREM1, TF, TRH, TRIM11, TRIAP1, TRIB3, TRPC1, TRPC5, TRPC6, PRSS1, PRSS2, TSC1, THBS1, TSPYL2, ZFP36, TWIST1, TXNIP, TYRO3, YY1, UBE2V1, UBE4A, UCHL1, UCP1, UCP2, UCP3, AXL, ULK1, UNC13B, PLAU, UMOD, UTRN, UTS2, AVPR2, VAC14, VASH1, VASH2, VAV1, VCAM1, VDAC1, VDAC2, VDR, VEGFA, VEGFB, VEGFC, FLT1, KDR, FLT4, HDLBP, VIM, VPS51, VSIG4, GC, VTN, VWF, WDR83, WFS1, WNK1, WT1, WWTR1, XBP1, XDH, XPR1, XRCC1, XYLT1, XYLT2, YAP1, YBX1, ZBTB16, ZEB1, ZEB2, ZNF236, ZNF410, SLC30A7, SLC30A8, ADRB1, AKAP7, AMOT, CAPG, CD46 |
| **440 JCYSTLF-related targets** |
| KDM4E, RAB9A, MAPT, NPC1, POLB, ATAD5, POLI, POLK, BRCA1, APEX1, IL2, NLRP3, STAT3, PTGS2, TP53, AR, JAK2, SORD, EGFR, GSR, CYP2C9, ALOX5, CDK4, CCNB1, AKR1B1, LCK, PTPN1, ADORA3, CTRB1, TYR, GLO1, TOP2B, CTRC, CDK5, CCNB2, CDK6, PIM1, PTGS2, CDK2, ALOX12, ALOX15, ALOX15B, PIK3R1, PLA2G1B, PDE5A, XDH, TOP1, TOP2A, PTGS1, AMY1A, ABCC2, CBR1, CBR3, HSPB1, HSF1, HSD17B1, HSD17B2, PLK1, CYP2C8, ESR1, HTR1A, PREP, DPP4, SRC, ABCC1, NR3C1, AR, CSNK2A1, BCHE, ACHE, PPARG, HIF1AN, GUSB, MAOA, MAOB, CA4, CA3, CA2, CA1, CA5A, CA5B, CA6, CA7, CA9, CA12, CA14, NEU2, DRD4, CYP19A1, BACE1, HSD11B1, CYP1A2, CYP1A1, CYP1B1, NOX4, CDK1, HSP90AB1, CHEK1, GSK3B, RPS6KB1, SGK1, AKT1, PDK1, PRKCA, MAPKAPK5, RPS6KA5, MAPKAPK2, MAPK13, MAPK12, MAPK11, MAPK14, MAPK8, MAPK1, CD38, LMNA, NFKB1, RECQL, GALK1, HSD17B10, KDM4E, KMT2A, SMN1, TSHR, TDP1, MAPT, CYP2C19, THRB, ALDH1A1, HIF1A, PMP22, USP2, STAT6, APEX1, BLM, HPGD, TP53, NPSR1, CYP2D6, CYP3A4, POLB, CALM1, ABCG2, GBA, DRD1, MPHOSPH8, EHMT2, CBX1, PIN1, ATAD5, FEN1, MAPK3, MAPK9, ABCB1, POLI, RGS4, POLH, POLK, SMAD3, USP1, UGT1A1, UGT1A10, UGT1A3, UGT1A4, UGT1A6, UGT1A8, UGT1A9, UGT2B15, PIK3CA, HSPA5, GPR35, MMP1, MMP2, MMP3, MMP9, MMP12, MMP13, CAMK2B, SLCO2B1, SLCO1B3, SLCO1B1, SYK, MPO, AVPR2, CXCR1, CCR4, ANTXR2, FLT3, PON1, PLAU, F2, KDR, PKN1, NEK6, NEK2, MET, MAP2K1, IGF1R, PTK2, AXL, AURKB, NUAK1, ALK, YES1, ARSA, MBNL1, EPHX2, PYGL, PIK3CG, MPG, PRKAA2, CAMK1D, CAMK1G, CAMK2A, CAMK2D, CAMK2G, CAMK4, CAMKK2, CDKL1, CHEK2, CLK1, CLK2, CLK3, CSNK1G1, CSNK1G2, CSNK1G3, DAPK3, DMPK, JAK1, MAP2K2, MAP2K6, MAP3K5, MAPK6, STK26, OXSR1, PAK4, PAK5, PAK6, CDK16, PDPK1, PIM2, PIM3, PLK4, PRKACA, RIOK2, RPS6KA3, SLK, STK10, STK16, STK17A, STK38, STK4, TNIK, PBK, VRK1, VRK2, VRK3, STK25, CYP2J2, PLG, DAPK1, AKR1B10, APP, SLC2A2, ABCB11, ELAVL1, ELAVL3, ABCC3, ABCC4, ELANE, RET, BRAF, LPCAT1, LPCAT2, NR1H3, HDAC6, KIT, ERBB2, HDAC2, TEK, HDAC8, HDAC1, RECQL, APEX1, MAPT, L3MBTL1, GAA, POLB, EIF4H, PABPC1, KDM4A, ALPG, ALPL, ALPI, VDR, FEN1, POLI, POLH, POLK, GMNN, WRN, TDP1, RAPGEF3, ADRB1, HTR1B, ADRB2, ABCB1, CYP2D6, ALB, ABCC2, SLC22A1, EHMT2, ALOX15B, DRD1, ADRB3, ATXN2, UGT1A1, UGT1A9, UGT2B10, UGT2B4, UGT2B7, ACHE, ADORA1, ADORA2A, ADORA3, ADRA1D, ADRA2A, ADRA2B, ADRA2C, SLC6A2, AGTR2, BDKRB2, CALCR, CNR1, CA2, CCR2, CCR4, CCR5, CXCR1, CXCR2, CCKAR, PTGS1, PTGS2, CYP1A2, CYP2A6, CYP2C19, CYP2C9, CYP2E1, CYP3A4, DRD2, DRD3, DRD4, SLC6A3, EDNRA, ESR1, ESR2, HRH1, HRH2, HMGCR, CYSLTR1, MC3R, MC4R, MC5R, MAOA, CHRM1, CHRM2, CHRM3, CHRM4, CHRM5, NPY1R, NPY2R, OPRD1, OPRK1, OPRM1, PDE5A, PTAFR, KCNH2, CASP1, CTSG, ELANE, MMP1, MMP9, PRKCA, MAPK3, MAPK1, MAPK14, PPP3CA, EGFR, FYN, ERBB2, LCK, PTPRC, HTR2A, HTR2B, HTR2C, HTR6, SLC6A4, SIGMAR1, FLT1, VIPR1, AVPR1A, SLC22A2, SLC10A1, SLCO2B1, SLCO1B3, SLCO1B1, TDP1, ARSA, SCN5A, CACNA1C, CYP2B6, CYP2C8, CYP2J2, ABCB11, AOX1, HTR1A, HTR7, ADRA1A, AGTR1, ABCC3, ABCC4, XDH, THRB, ALDH1A1, CYP3A4, ABCG2, ABCB1, CBX1, USP1, SLCO2B1, TDP1, TARDBP, SLCO1B1, SLCO1B3, RAPGEF4, EGFR, TSHR, GLA, TP53, MAPK1, HIF1A. |
| **92 common targets** |
| HTR2A, ADRA2C, CCR2, CCR5, DRD2, EDNRA, IGF1R, MAP3K5, MAPK12, MMP1, ADORA1, PRKAA2, AGTR1, AGTR2, AKT1, ALB, AKR1B1, APEX1, HSPA5, BDKRB2, CD38, CDK5, CNR1, CYP19A1, CXCR1, CXCR2, SORD, DPP4, DRD3, EGFR, ELAVL1, ESR1, PTK2, GSR, GSK3B, SLC2A2, HDAC2, HIF1A, HSPB1, EPHX2, IL2, JAK1, JAK2, CAMKK2, LMNA, ALOX12, MAPK1, MAPK3, MAPK8, MAPK14, MMP12, MMP2, MMP3, MMP9, MAP2K2, NFKB1, NLRP3, NOX4, NR1H3, NUAK1, TP53, PIK3R1, PLA2G1B, PDE5A, PDK1, MPO, PLG, PON1, PPARG, PTAFR, SLC22A2, SGK1, SMAD3, STAT3, AXL, PLAU, AVPR2, VDR, FLT1, KDR, XDH, ADRB1, PTGS1, PTGS2, PIN1, PIK3CA, PIK3CG, KMT2A, PRKCA, SYK, LCK, GLO1. |

| **Table S3: Network parameters of the core targets of JCYSLTF in the treatment of DN** | | | | | | | |
| --- | --- | --- | --- | --- | --- | --- | --- |
| **Gene** | **Degree** | **Betweenness** | **Closeness** | **Gene** | **Degree** | **Betweenness** | **Closeness** |
| AKT1 | 64 | 1167.0233 | 0.7711864 | ADORA1 | 5 | 16.43592 | 0.4764398 |
| HIF1A | 60 | 921.78735 | 0.74590164 | MMP3 | 21 | 15.27284 | 0.5481928 |
| ALB | 59 | 816.74225 | 0.733871 | LCK | 23 | 14.220113 | 0.55151516 |
| EGFR | 51 | 499.30698 | 0.68939394 | DRD2 | 6 | 13.737747 | 0.47395834 |
| PPARG | 46 | 458.25702 | 0.6691176 | DPP4 | 12 | 11.4900875 | 0.5141243 |
| TP53 | 51 | 377.74963 | 0.68421054 | PLAU | 17 | 11.09748 | 0.53216374 |
| STAT3 | 47 | 352.83282 | 0.6691176 | BDKRB2 | 4 | 10.217866 | 0.3939394 |
| MAPK3 | 50 | 318.63788 | 0.67407405 | LMNA | 14 | 10.037663 | 0.5229885 |
| PTGS2 | 43 | 293.20792 | 0.65 | CDK5 | 9 | 9.60198 | 0.49456522 |
| CNR1 | 10 | 246.44116 | 0.50276244 | SYK | 17 | 9.236295 | 0.5260116 |
| GSR | 16 | 195.51627 | 0.5290698 | CD38 | 14 | 8.484891 | 0.51704544 |
| MAPK14 | 33 | 191.02197 | 0.59477127 | ELAVL1 | 10 | 6.144903 | 0.50555557 |
| KDR | 33 | 183.7565 | 0.59090906 | ADRA2C | 3 | 6.0621824 | 0.37603307 |
| SLC2A2 | 7 | 180.22223 | 0.4918919 | HSPA5 | 17 | 5.8843894 | 0.53216374 |
| PRKCA | 22 | 138.1989 | 0.55151516 | NLRP3 | 15 | 5.847315 | 0.5141243 |
| AGTR1 | 16 | 108.89222 | 0.5229885 | PTGS1 | 6 | 5.810885 | 0.4715026 |
| MMP9 | 39 | 104.46687 | 0.61904764 | FLT1 | 16 | 5.028076 | 0.52 |
| JAK2 | 33 | 87.954636 | 0.59090906 | SORD | 5 | 4.639313 | 0.44174758 |
| PIK3CA | 31 | 84.68174 | 0.572327 | MAP3K5 | 13 | 4.351668 | 0.50276244 |
| GSK3B | 25 | 78.27823 | 0.5582822 | MAPK12 | 12 | 4.132951 | 0.50555557 |
| MAPK1 | 35 | 74.76294 | 0.6066667 | GLO1 | 5 | 3.6258657 | 0.38396624 |
| HSPB1 | 18 | 73.18211 | 0.53846157 | MMP1 | 19 | 3.534433 | 0.53846157 |
| MPO | 24 | 71.48626 | 0.5582822 | XDH | 7 | 3.3168323 | 0.45728642 |
| EDNRA | 8 | 70.63554 | 0.48404256 | ALOX12 | 5 | 3.222784 | 0.4212963 |
| ESR1 | 36 | 68.46831 | 0.6107383 | PIN1 | 8 | 2.5881865 | 0.48924732 |
| IL2 | 34 | 65.48462 | 0.59477127 | CXCR2 | 14 | 2.2545888 | 0.5229885 |
| PTK2 | 27 | 63.83179 | 0.572327 | PDK1 | 4 | 1.853438 | 0.4642857 |
| CCR5 | 16 | 52.44064 | 0.5141243 | AGTR2 | 5 | 1.7215693 | 0.43961352 |
| PLG | 25 | 51.817257 | 0.5617284 | CAMKK2 | 3 | 1.354506 | 0.3855932 |
| MMP2 | 31 | 51.781845 | 0.58709675 | PON1 | 6 | 1.2808303 | 0.455 |
| AKR1B1 | 11 | 49.65394 | 0.5083799 | HTR2A | 3 | 0.8778062 | 0.43961352 |
| MAPK8 | 30 | 44.049843 | 0.5833333 | EPHX2 | 2 | 0.8095238 | 0.35686275 |
| APEX1 | 12 | 41.592247 | 0.50276244 | MMP12 | 7 | 0.65 | 0.45273632 |
| PIK3R1 | 25 | 40.434834 | 0.5416667 | CXCR1 | 10 | 0.5644231 | 0.4918919 |
| CCR2 | 16 | 39.350735 | 0.51704544 | CYP19A1 | 10 | 0.50576764 | 0.50555557 |
| SMAD3 | 31 | 38.351624 | 0.57961786 | AXL | 9 | 0.1 | 0.47395834 |
| HDAC2 | 13 | 31.619467 | 0.51704544 | VDR | 9 | 0.0952381 | 0.5 |
| MAP2K2 | 17 | 30.56332 | 0.51123595 | PTAFR | 3 | 0 | 0.41363636 |
| NOX4 | 18 | 29.552961 | 0.5449102 | KMT2A | 2 | 0 | 0.42723006 |
| PIK3CG | 18 | 26.778711 | 0.52 | NR1H3 | 2 | 0 | 0.455 |
| IGF1R | 28 | 25.354626 | 0.572327 | PDE5A | 2 | 0 | 0.44390243 |
| PLA2G1B | 11 | 24.047655 | 0.5 | AVPR2 | 2 | 0 | 0.41363636 |
| PRKAA2 | 7 | 22.19048 | 0.46907216 | ADRB1 | 2 | 0 | 0.45273632 |
| JAK1 | 24 | 20.93585 | 0.5582822 | SLC22A2 | 1 | 0 | 0.3309091 |
| SGK1 | 7 | 19.599977 | 0.45273632 | NUAK1 | 1 | 0 | 0.42523363 |
| NFKB1 | 25 | 18.966156 | 0.55487806 | DRD3 | 1 | 0 | 0.33579335 |

| **Table S4: KEGG enrichment analysis** | | | | |
| --- | --- | --- | --- | --- |
| **ID** | **Pathway** | **Rich factor** | **pvalue** | **Count** |
| hsa04015 | HIF-1 signaling pathway | 0.157303371 | 7.77E-10 | 15 |
| hsa04066 | cell senescence | 0.146067416 | 1.70E-08 | 23 |
| hsa04668 | TNF signaling pathway | 0.168539326 | 5.90E-09 | 13 |
| hsa04931 | Insulin resistance | 0.112359551 | 1.44E-08 | 10 |
| hsa04210 | Apoptosis-related signaling pathway | 0.123595506 | 3.13E-08 | 11 |
| hsa04140 | Autophagy-related signaling pathway | 0.123595506 | 1.55E-07 | 16 |
| hsa05415 | Diabetic cardiomyopathy | 0.157303371 | 2.13E-07 | 14 |
| hsa04151 | PI3K-Akt signaling pathway | 0.247191011 | 2.22E-07 | 22 |
| hsa04620 | Toll-like receptor signaling pathway | 0.112359551 | 3.08E-07 | 10 |
| hsa04910 | Insulin signaling pathway | 0.101123596 | 8.55E-07 | 9 |
| hsa04218 | AMPK signaling pathway | 0.123595506 | 9.17E-06 | 11 |
| hsa04930 | Type II diabetes mellitus | 0.06741573 | 1.61E-05 | 11 |
| hsa04152 | Rap1 signaling pathway | 0.08988764 | 4.68E-05 | 14 |
| hsa04630 | JAK-STAT signaling pathway | 0.08988764 | 0.000357351 | 8 |
| hsa04064 | NF-kappa B signaling pathway | 0.06179775 | 0.005350999 | 15 |

| **Table S5: GO enrichment analysis** | | | | | |
| --- | --- | --- | --- | --- | --- |
| **ID** | **Description** | **Rich factor** | **pvalue** | **Count** | **class** |
| GO:0001666 | response to hypoxia | 0.152173913 | 3.36E-14 | 14 | BP |
| GO:0034599 | cellular response to oxidative stress | 0.239130435 | 9.41E-10 | 22 | BP |
| GO:0007568 | senescence | 0.163043478 | 7.54E-12 | 15 | BP |
| GO:0010506 | regulation of autophagy | 0.152173913 | 7.14E-12 | 14 | BP |
| GO:0008631 | intrinsic apoptotic signaling pathway | 0.065217391 | 6.05E-13 | 6 | BP |
| GO:0005925 | focal adhesion | 0.141304348 | 7.26E-08 | 13 | CC |
| GO:0005924 | cell-substrate adherens junction | 0.141304348 | 7.70E-08 | 13 | CC |
| GO:0030055 | cell-substrate junction | 0.141304348 | 8.90E-08 | 13 | CC |
| GO:0098978 | glutamatergic synapse | 0.130434783 | 1.56E-07 | 12 | CC |
| GO:0005912 | adherens junction | 0.141304348 | 9.21E-07 | 13 | CC |
| GO:0019902 | phosphatase binding | 0.163043478 | 3.37E-10 | 15 | MF |
| GO:0004713 | protein tyrosine kinase activity | 0.130434783 | 2.01E-12 | 12 | MF |
| GO:0019903 | protein phosphatase binding | 0.119565217 | 1.30E-10 | 11 | MF |
| GO:0004879 | nuclear receptor activity | 0.054347826 | 4.83E-06 | 5 | MF |
| GO:0098531 | transcription factor activity | 0.054347826 | 4.83E-06 | 5 | MF |

| **TableS6: GO enrichment analysis of senescence-related targets** | | | | |
| --- | --- | --- | --- | --- |
| **Term** | **count** | **enrichment** | **pvalue** | **class** |
| Autophagy process | 12 | 1.303383913 | 5.27E-10 | BP |
| Intracellular signal transduction | 8 | 1.260340435 | 5.60E-09 | BP |
| Cellular response to stress | 10 | 1.244253478 | 5.27E-09 | BP |
| Regulation of catalytic activity | 9 | 1.293383913 | 5.27E-09 | BP |
| Cellular response to tumor necrosis factor | 7 | 1.186427391 | 1.87E-08 | BP |
| MAP kinase activity | 4 | 1.262514348 | 1.08E-06 | MF |
| MAP kinase kinase activity | 4 | 1.262514348 | 1.11E-06 | MF |
| Insulin receptor substrate binding | 2 | 1.262514348 | 0.0042 | MF |
| Protein serine/threonine/tyrosine kinase activity | 5 | 1.251644783 | 4.81E-07 | MF |
| Mitogen-activated protein kinase binding | 2 | 1.262514348 | 0.0185 | MF |
| Phosphatidylinositol 3-kinase complex, class IA | 2 | 1.284253478 | 0.015 | CC |
| Pseudopodium | 2 | 1.251644783 | 0.0222 | CC |
| Cell-cell junction | 4 | 1.240775217 | 0.0366 | CC |
| Mitochondrion | 9 | 1.175557826 | 0.00013 | CC |
| Anchoring junction | 6 | 1.175557826 | 0.0261 | CC |

| **TableS7**：**KEGG enrichment analysis of senescence-related targets** | | | |
| --- | --- | --- | --- |
| **Term** | **count** | **enrichment** | **pvalue** |
| VEGF signaling pathway | 8 | 2.34 | 2.09E-16 |
| Relaxin signaling pathway | 9 | 2.03 | 3.30E-16 |
| Apoptosis | 8 | 1.97 | 5.81E-14 |
| Autophagy- animal | 2 | 1.91 | 7.16E-12 |
| Choline metabolism in cancer | 7 | 2.05 | 9.44E-10 |
| MAPK signaling pathway | 8 | 1.63 | 1.56E-11 |
| PI3K-Akt signaling pathway | 8 | 1.54 | 6.69E-11 |
| Ras signaling pathway | 7 | 1.67 | 2.25E-10 |
| Insulin signaling pathway | 6 | 1.84 | 8.35E-10 |
| mTOR signaling pathway | 6 | 1.78 | 1.68E-09 |
| Insulin resistance | 4 | 1.76 | 2.10E-06 |
| AMPK signaling pathway | 4 | 1.7 | 3.33E-06 |
| TGF-beta signaling pathway | 3 | 1.7 | 8.26E-05 |
| JAK-STAT signaling pathway | 3 | 1.46 | 0.00038 |
| Mitophagy - animal | 2 | 1.68 | 0.0022 |
| Cell cycle | 2 | 1.4 | 0.0069 |
