# Supplementary material for: Network analysis to explore the anti-senescence mechanism of Jinchan Yishen Tongluo Formula (JCYSTLF) in diabetic kidneys
Source: Heliyon. 2024 Apr 12;10(9):e29364. doi: 10.1016/j.heliyon.2024.e29364 (PMC11076649; doi:10.1016/j.heliyon.2024.e29364)

Fig.S2-Fig.5A-LC3B

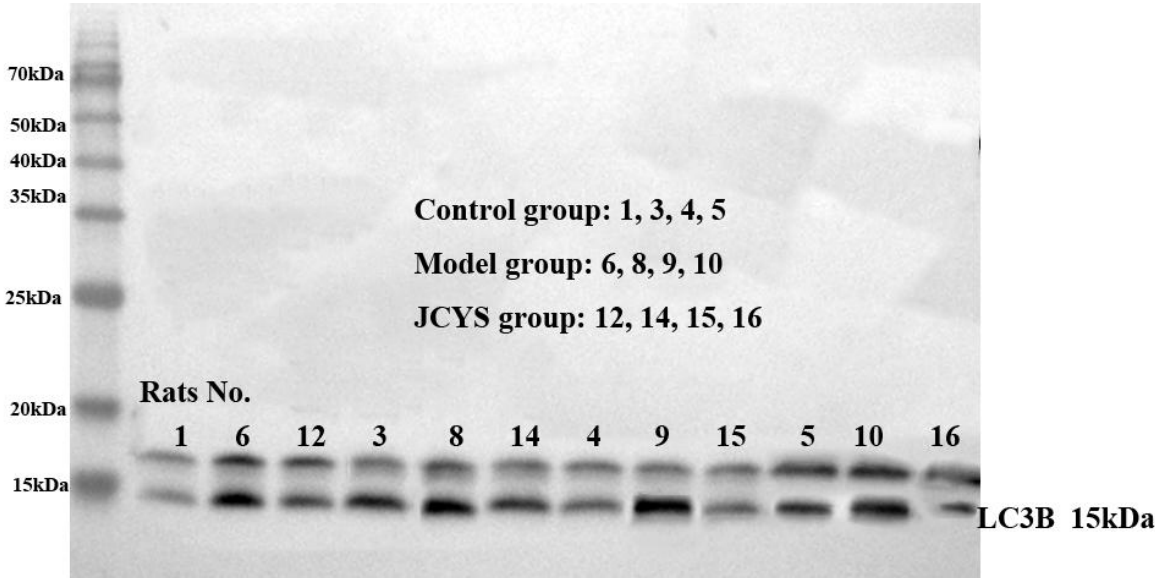

Fig.S3-Fig.5A-p62

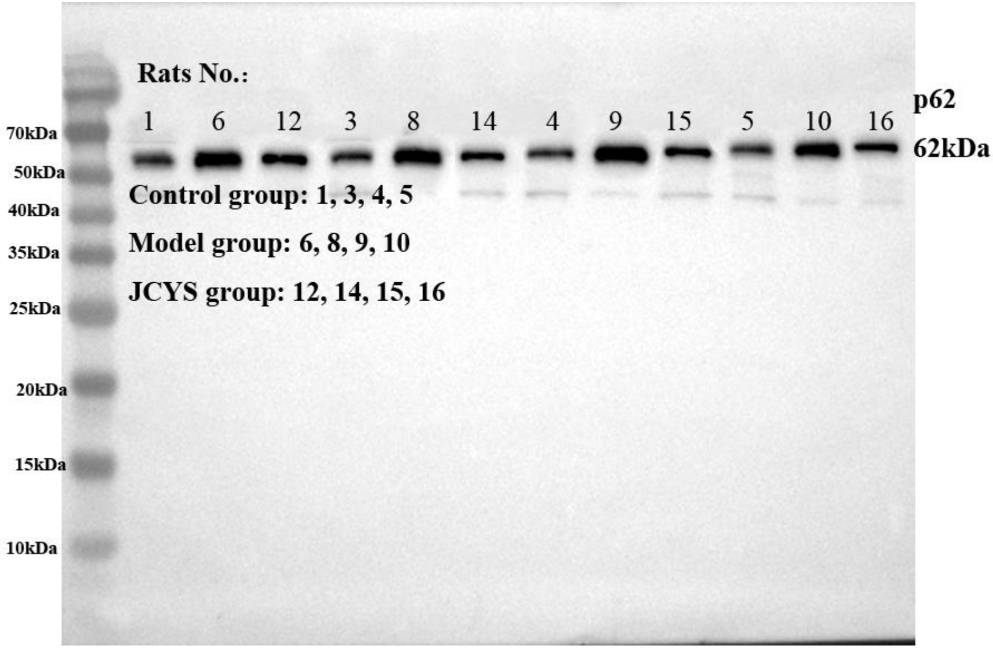

Fig.S4- Fig.5A-β-actin

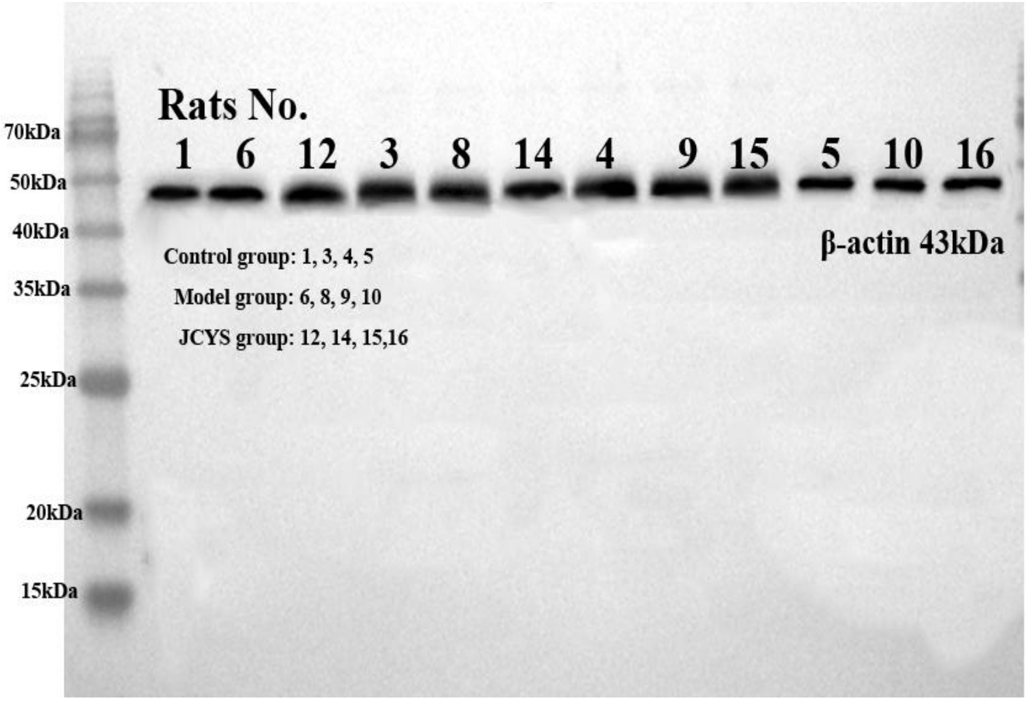

Fig.S5- Fig.5B-p53

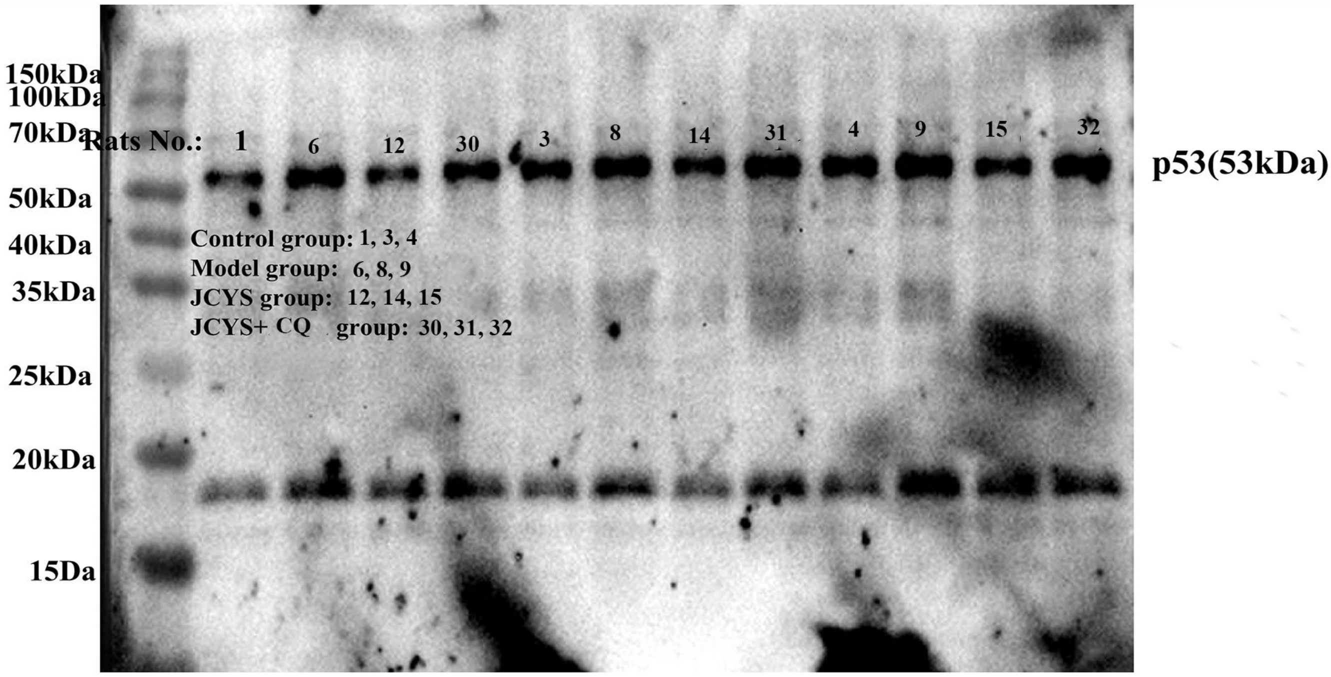

Fig.S6- Fig.5B-β-p21

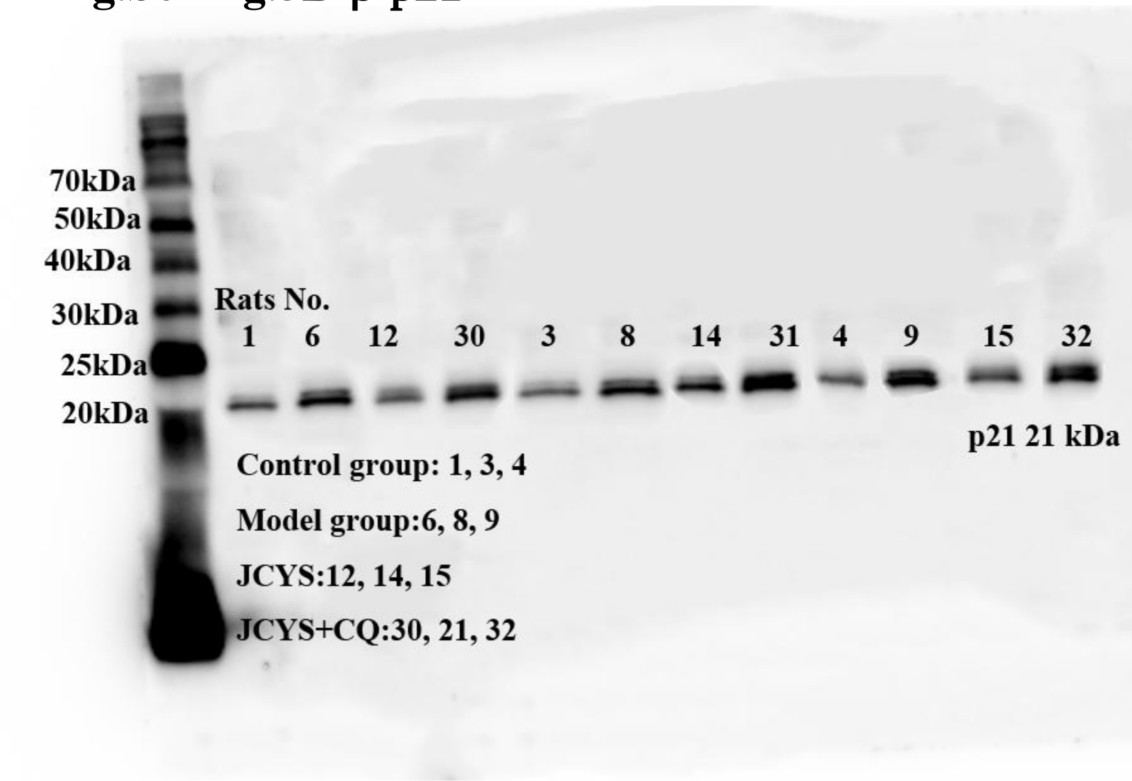

Fig.S7- Fig.5B-β-actin

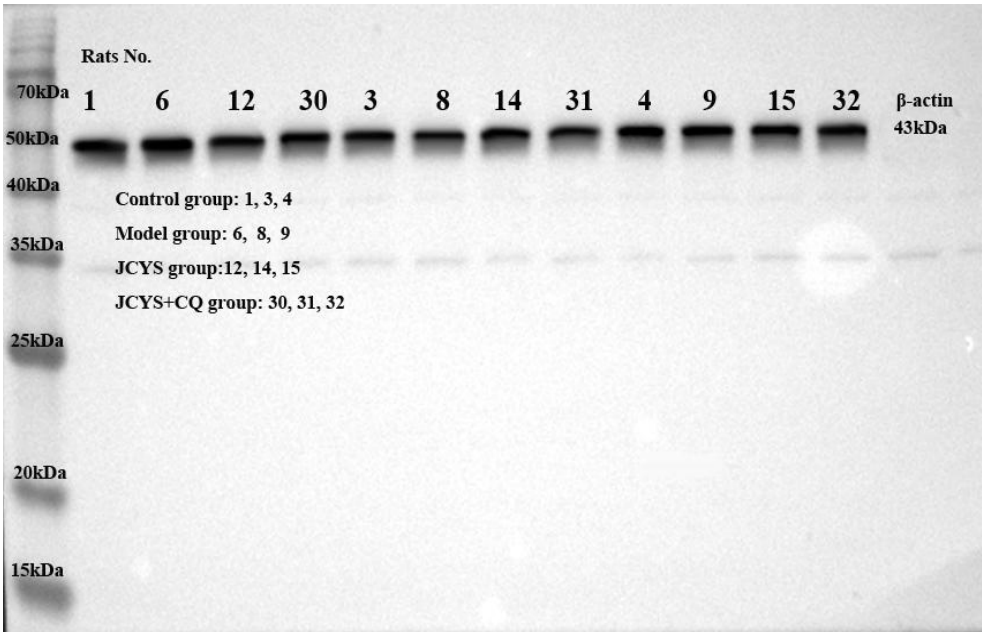

Fig.S8- Fig.6-HIF-1α

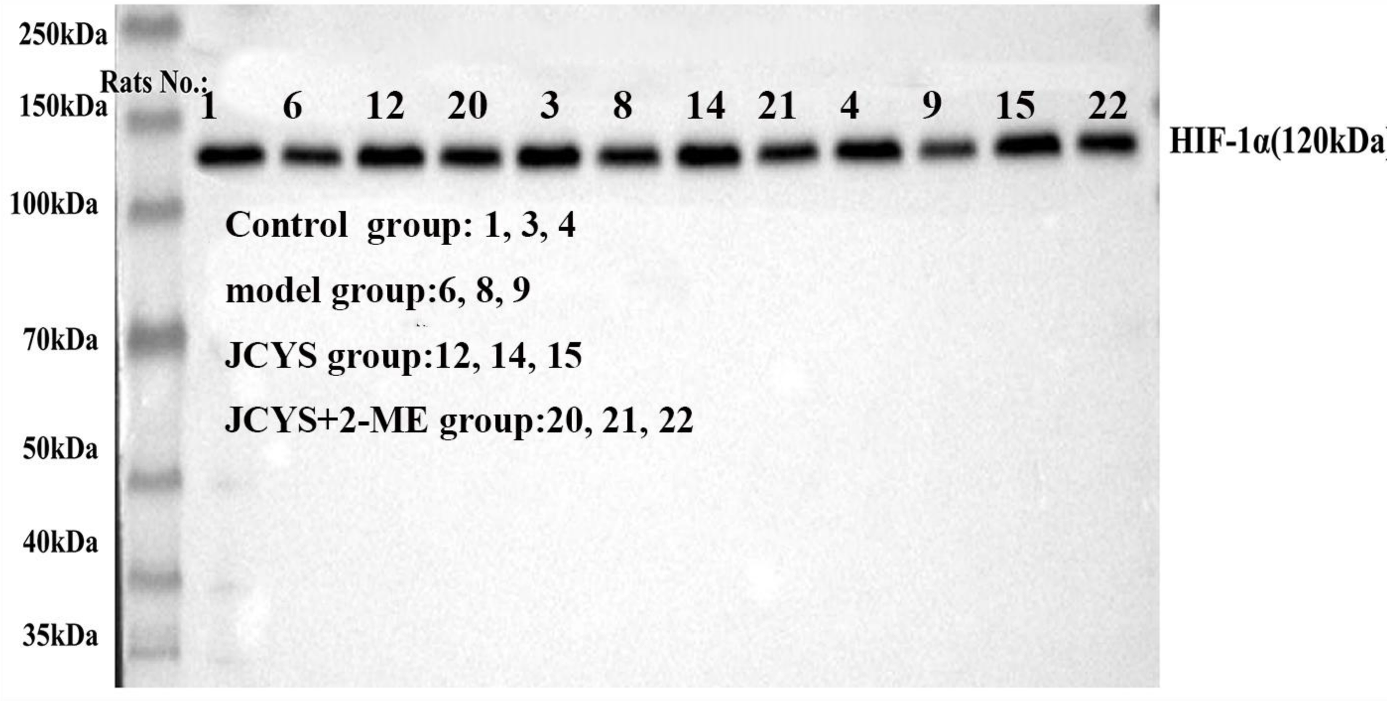

Fig.S9- Fig.6-p62

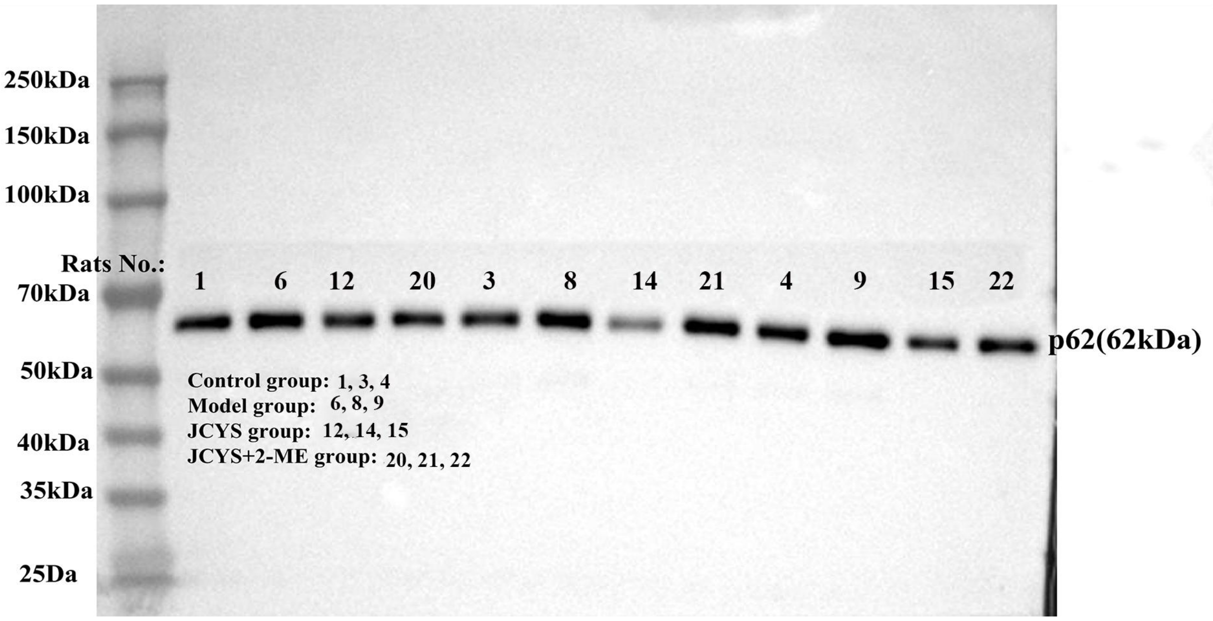

Fig.S10-Fig.6-LC3B

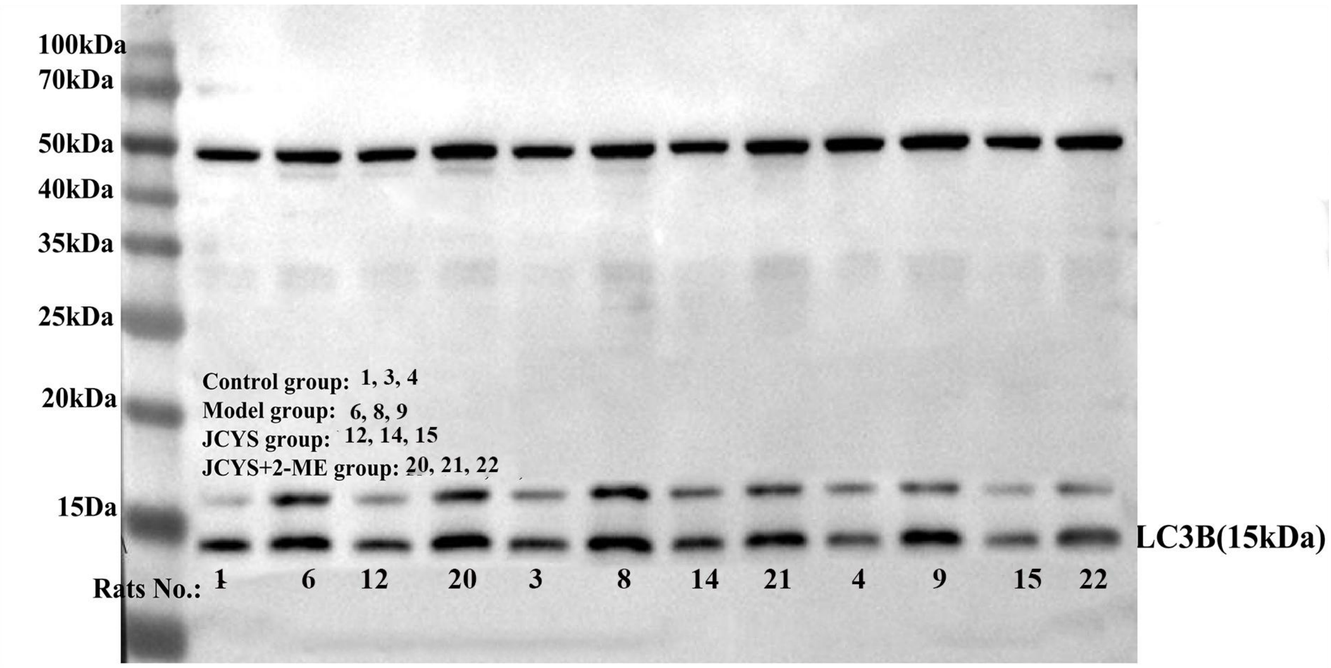

Fig.S11-Fig.6-β-actin

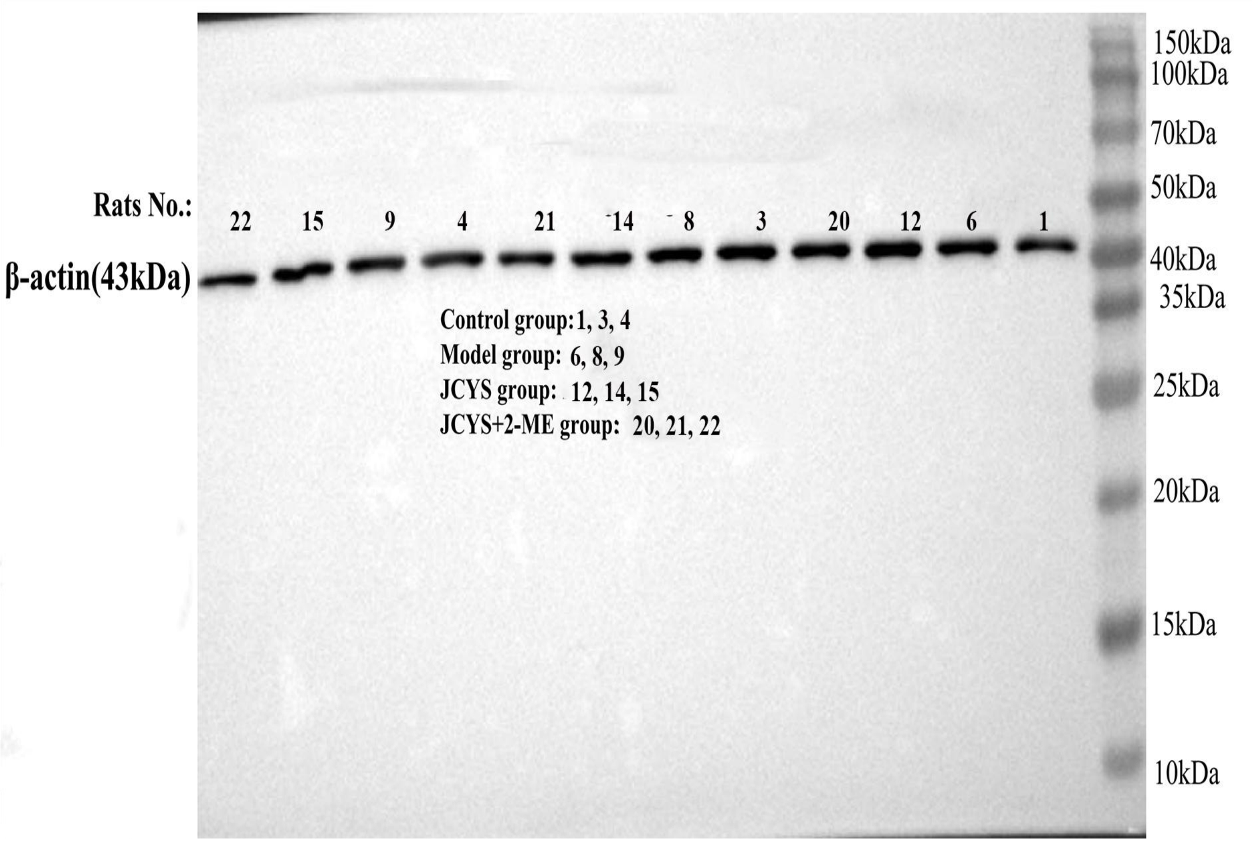

Fig.S12-Fig.7-p53

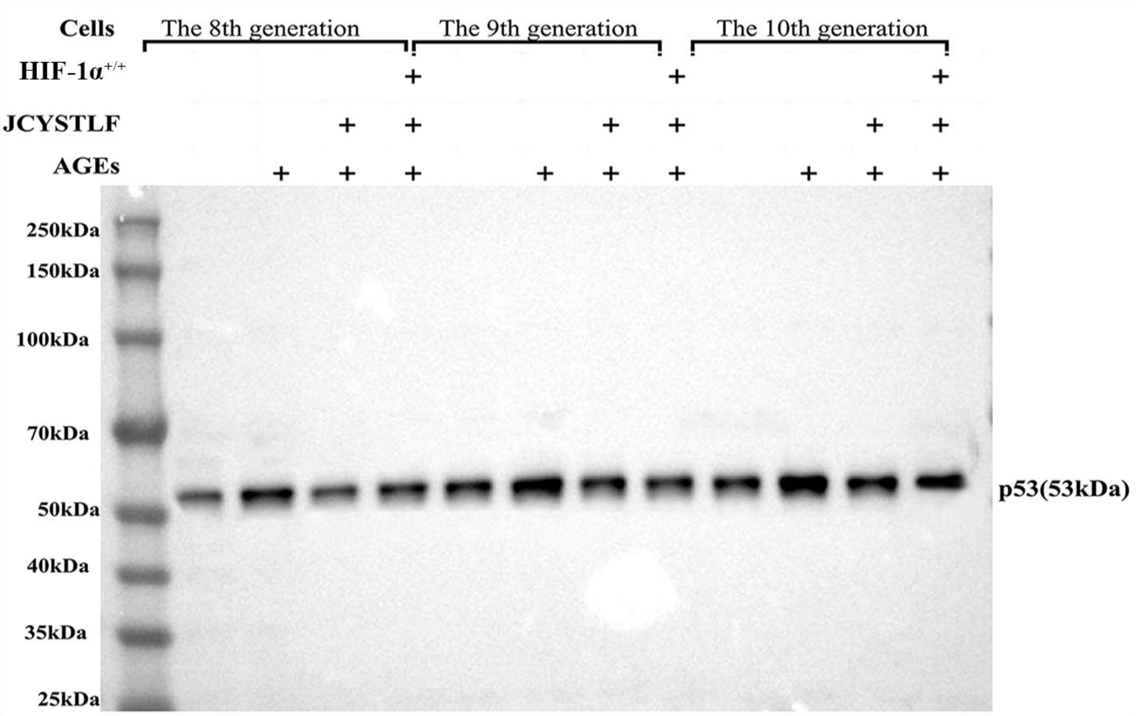

Fig.S13-Fig.7-HIF-1α

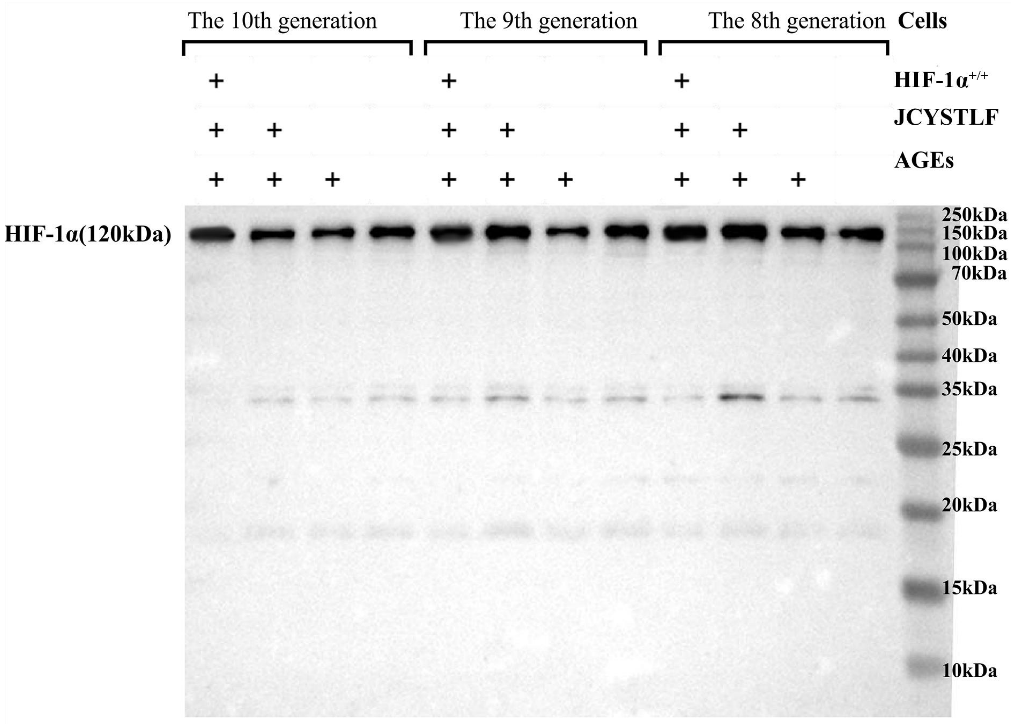

Fig.S14-Fig.7-LC3B

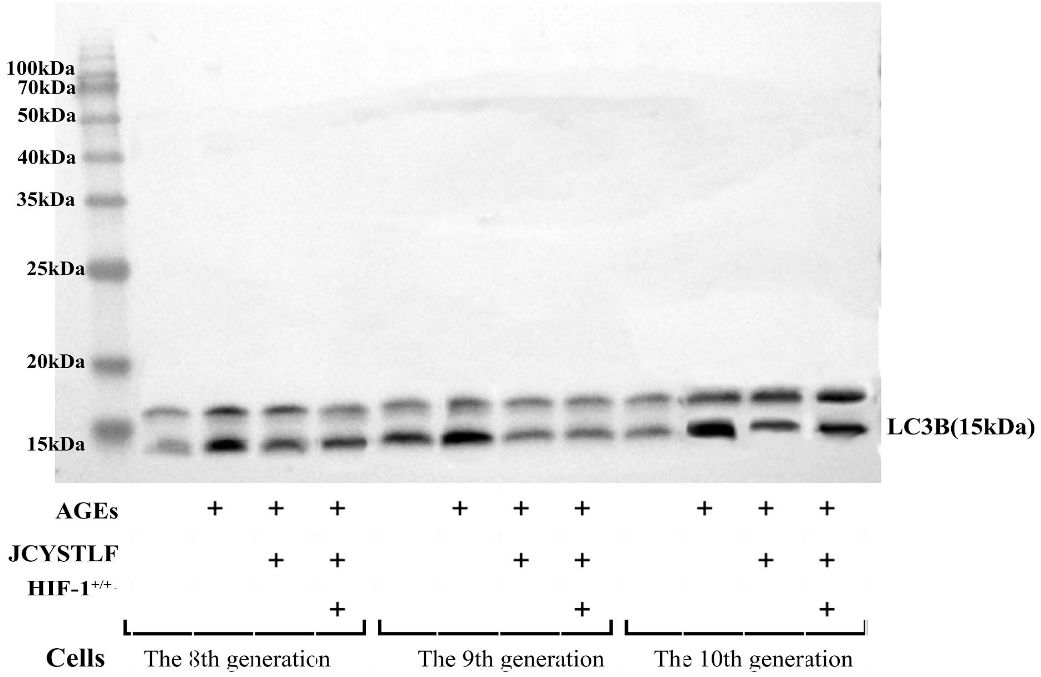

Fig.S15-Fig.7-p21

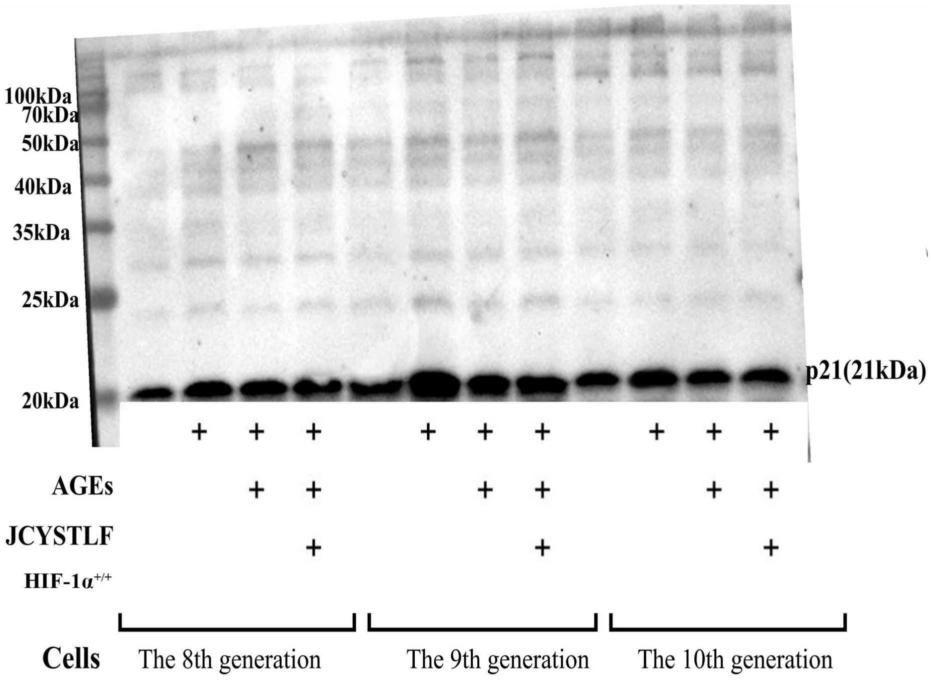

Fig.S16-Fig.7-p62

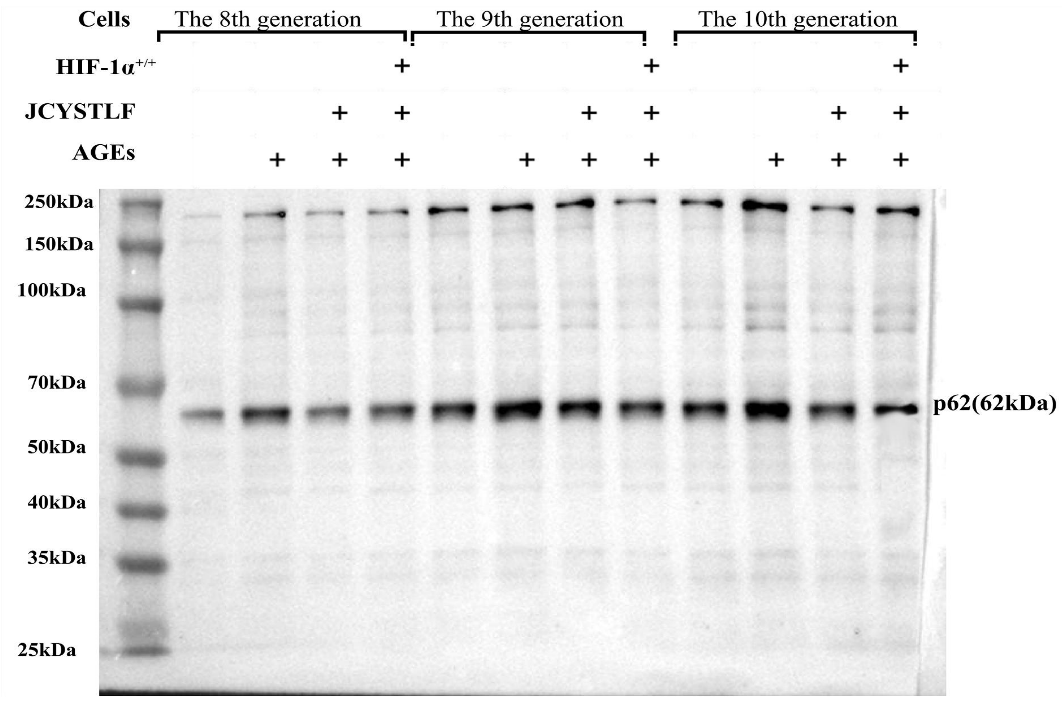

Fig.S17-Fig.7-β-actin

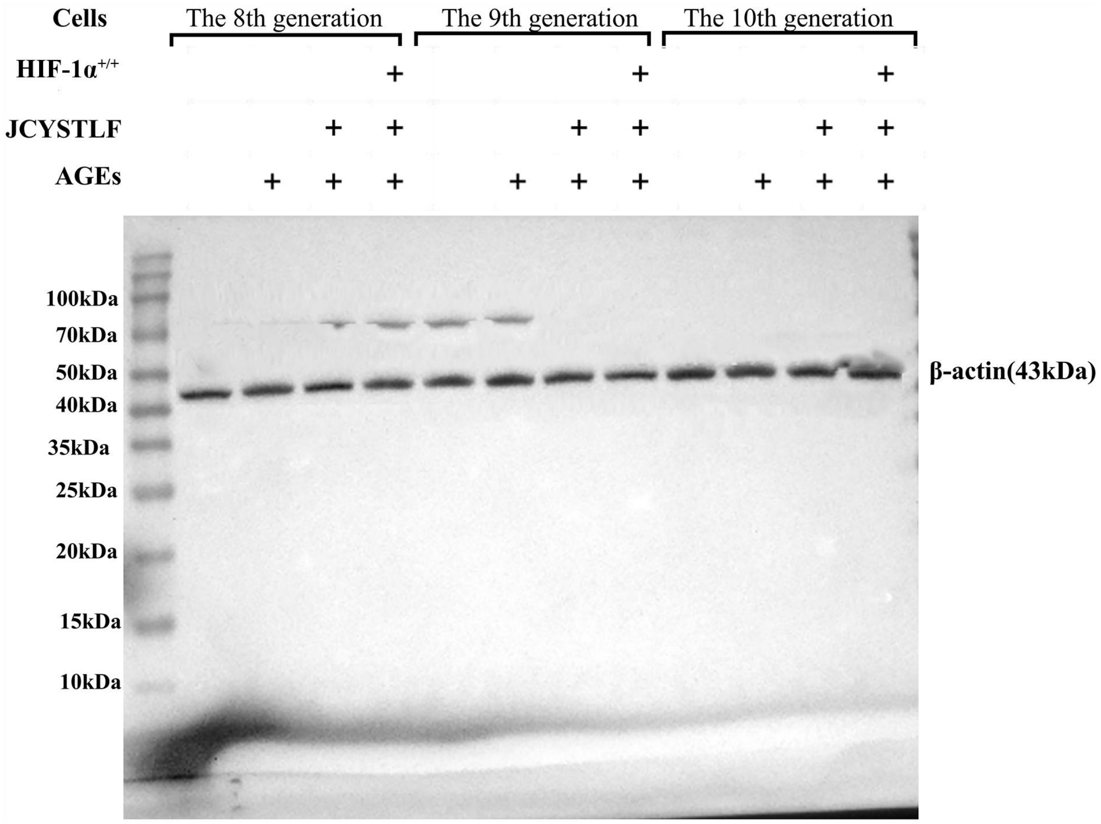

Supplement: Multimedia component 3 [file mmc3.pdf]
